# Sodium–calcium exchanger isoform-3 targeted *Withania somnifera* (L.) Dunal therapeutic intervention ameliorates cognition in the 5xFAD mouse model of Alzheimer’s disease

**DOI:** 10.1038/s41598-022-05568-2

**Published:** 2022-01-27

**Authors:** Henok Kessete Afewerky, Hao Li, Tongmei Zhang, Xinyan Li, Yacoubou Abdoul Razak Mahaman, Limin Duan, Pengwei Qin, Jiequn Zheng, Lei Pei, Youming Lu

**Affiliations:** 1 Department of Neurobiology, School of Basic Medicine, Tongji Medical College, Huazhong University of Science and Technology, Wuhan, China; 2 Department of Pathology and Pathophysiology, School of Basic Medicine, Tongji Medical College, Huazhong University of Science and Technology, Wuhan, China; 3 School of Allied Health Professions, Asmara College of Health Sciences, Asmara, Eritrea; 4 The Institute for Brain Research, Collaborative Innovation Center for Brain Science, Huazhong University of Science and Technology, Wuhan, China; 5 Department of Physiology, School of Basic Medicine, Tongji Medical College, Huazhong University of Science and Technology, Wuhan, China

**Keywords:** Neuroscience, Diseases, Medical research

## Abstract

The third isoform of the Na^+^–Ca^2+^ exchanger (NCX3) is crucial for a physiological fine-tuning of the Ca^2+^ fluxes in excitable tissues. In this view, the NCX3 accounts for the aberrant Ca^2+^ influx seen during neuronal excitotoxicity, such as in Alzheimer’s disease (AD). However, little is known about NCX3 regulation and functional properties. *Withania somnifera* (L.) Dunal (*W. somnifera*), a traditional indigenous plant widely recognized for having numerous medicinal values, was undertaken to determine its potential therapeutic benefit against aggregated Aβ_1–42_-induced NCX3 dysregulation and the thereof cognition impairment in 5xFAD mice. The undertaken sourced dried roots of authenticated *W. somnifera* physicochemical compositional tests satisfied standards of pharmacognostic quality, and further phytochemical analysis of the roots methanol extract revealed the roots constitute several antioxidants. Following an intra-gastric gavage administration of synthesized *W. somnifera* roots methanolic extract from postnatal day 30 (P30) to P75, in vivo cognitional studies and then neurochemical examinations of the NCX3 expression level, Aβ plaque deposition, and antioxidant activities in the AD-associated brain regions of 4-month-old 5xFAD mice suggests that the oxidative stress normalizing effects of *W. somnifera* constituents, operating on the NCX3, may have a therapeutic role in the improvement of cognition in AD.

## Introduction

The Na^+^–Ca^2+^ exchanger (NCX) is a plasma membrane transporter that facilitates both Ca^2+^ and Na^+^ fluxes in a bidirectional way^1^ with a stoichiometry of 3 Na^+^ ions for every Ca^2+^ ion. Depending on the intracellular levels of Na^+^ and Ca^2+^, NCX operates in either forward (Ca^2+^ efflux) or reverse (Ca^2+^ influx) mode using the Na^+^ gradient across the plasma membrane as a source of energy^2^. In this view, NCX protects cells from Ca^2+^ overload and eventual death^3^. Three isoforms of the NCX, namely NCX1, NCX2, and NCX3, encoded by three distinct genes, have been determined in mammals^4^. These isoforms share about 70% structural similarities, given the fact that NCX3 possesses a 73% sequence identity with NCX1 and 75% sequence identity with NCX2^5^. Molecular aspects of these isoforms have been extensively studied, including the insights on the role of NCX3 in Aβ_1–42_ induced neurodegeneration, such as Alzheimer’s disease (AD). These insights have been recently reported and are therefore not covered here in any detail^3,6^. Generally speaking, NCX3 plays a significant role in neurons, where an alteration in cytosolic Ca^2+^ concentration represents critical events in several physiological (e.g. synaptic transmission) and pathological phenomenon (e.g. AD)^3,6,7^. However, little is known about the possible therapeutic interventions that target NCX3 regulation and functional properties.

Various plants and herbal preparations that have shown promise in dementia symptomatic improvement have been of research interest. However, the unavailability of a dementedness-modifying drug, due to the underlying pathologies complexity, necessitates a need to develop a different target-based possible therapeutic agent strategy. *Withania somnifera* (L.) Dunal (*W. somnifera*) belongs to the plant family Solanaceae, and it is indigenous to the tropical and subtropical regions of the world^8,9^. Different parts of the plant, predominantly roots, have been traditionally used for therapeutic purposes against neurological disorders since antiquity^9–11^. Of note, *W. somnifera* acute and sub-acute toxicity studies in rodents administrated with oral^12,13^ or intraperitoneal injections^14,15^ found it non-toxic even at 2000 mg/kg and 1100 mg/kg body weight, respectively. Owing to its pronounced stress-busting qualities, the plant has been given its species name somnifera, a Latin word meaning ‘sleep-inducer’^11,16^. *W. somnifera* exerts multiple neuroprotective pharmacologic actions, such as decreasing oxidative stress by restoring antioxidant levels^17^, modulating mitochondrial function^18^, regulating apoptosis^19^, reducing inflammation^20^, clearance of Aβ levels^21^, and attenuating synaptic and dendritic loss^22^. Furthermore, previous studies have shown that methanol extract of *W. somnifera* induces memory consolidation in hippocampal CA1 neurons^23^ and the extract putative pharmacological compounds, such as Withaferin A, Withanolide A, Withanolide B, and Withanolide D, are determined to reduce cocaine-induced Aβ levels^24^, augment glycine site of N-methyl-D-aspartate (NMDA) receptor^23^, prevent NMDA-induced excitotoxicity^25^, and inhibit GluN2B containing NMDARs through allosteric mode similar to the well-known selective antagonist Ifenprodil^26^. Despite these copious reported benefits, the possible effect of *W. somnifera* on the NCX3 expression level in Aβ-induced neurodegeneration has not been reported.

Given the above-mentioned therapeutic value of *W. somnifera* against neurological diseases, Alzheimer’s disease (AD) is notably albeit far less well studied. AD is a chronic neurodegenerative disease that represents 60–80% of diagnosed cases of age-related dementia^27^. To elucidate the AD underlying pathophysiology and pharmacologically address the issue, several research groups generated manifold widely used amyloid-based AD mouse models on multiple inbred and hybrid backgrounds. From these murine, 5xFAD mice express human APP and PSEN1 transgenes with a total of five AD-linked mutations, namely, APP with K670N/M671L (Swedish mutation^28^), I716V (Florida mutation^29^), and V717I (London mutation^30^), and PSEN1 with M146L and L286V mutations^31^ to predominantly make Aβ_1–42_. Thence, 5xFAD mice represent a very aggressive amyloid deposition model that develops intraneuronal Aβ_1–42_ at 1.5 months, plaques at 2 months, memory deficits at 4 months, and neuron loss at 9 months of age^32^. These characteristics make 5xFAD mice a robust model for investigating the preventive role of *W. somnifera* against aggregated Aβ_1–42_-induced NCX3 dysregulation in AD.

Taking these previous studies together, it is plausible that *W. somnifera* may improve cognition via regulation of NCX3 in restoring baseline Ca^2+^ levels following depolarization in neurons. Thus, for the first time, this study investigated the effects of *W. somnifera* roots methanolic extract against Aβ_1–42_-mediated NCX3 dysregulation and the thereof cognition impairment in 5xFAD, a well-studied mouse model of AD.

## Materials and methods

### Reagents

All reagents used in the present study were purchased from Abcam (Wuhan, Hubei, China) unless otherwise stated.

### *W. somnifera* roots

Authenticated *W. somnifera* dried roots were sourced from a herbarium indexed at NYBG Steere Herbarium (IBSC799032, IBSC, Guangzhou, China) and the plant name was checked with World Flora Online (WFO) database^33^. This study’s experimental research using *W. somnifera* roots complied with the relevant institutional, national, and international guidelines and legislation in studies on plants. The dried plant roots were powdered coarsely using a pulverizer and passed through a 38 µm sieve. From the sieved material, 0.1% was used for compositional tests of ascertaining pharmacognostic standards^34^ to estimate the physicochemical constitution of the roots, including total sugar, total protein, and total lipid, using standard methods^35^. Moreover, loss on drying, total ash, water soluble ash, acid insoluble ash, water soluble extractive value, and alcohol soluble extractive value were determined as described in the Ayurvedic Pharmacopoeia of India^34^.

### Preparation of *W. somnifera* methanol extract

The roots powder was soaked in methanol (25 g/250 ml) for 24 h withheld in a gently shaking apparatus at room temperature for extraction. The extract was filtered with a Whatman filter paper (125 mm; 1441–125, GE Healthcare Life Sciences, UK) while excess solvent was allowed to evaporate under reduced pressure at 40 °C using a rotary vacuum evaporator. Following to determining the mean yield as a mass of the obtained extract per 100 g of *W. somnifera* roots, the filtrate was stored in a refrigerator at 4 °C and used throughout the study.

### Phytochemical screening

Qualitative phytochemical studies of the extract (Table 1) were performed as per standard procedures^36–38^ to determine the presence of biochemically active constituent organic compounds. In brief, to test for the presence of alkaloids compound, few drops of iodine solution in potassium iodide were added to 2 ml of the extract solution. Formation of reddish-brown precipitate confirms the presence of alkaloids. To test for flavonoids, 1 ml of the extract was treated with 0.5 ml of concentrated hydrochloric acid and then warmed on a water bath for 15 min. An intense red color signifies the presence of flavonoids. To test for glycosides, 50 mg of the extract was hydrolyzed with concentrated hydrochloric acid for 2 h on a water bath, filtered, and then 2 ml of the filtered hydrolysate was thoroughly mixed with 3 ml chloroform. A pink color signifies the presence of glycosides. To test for phenols, the extract (50 mg) was dissolved in 5 ml of distilled water, and to this 3 ml of 10% lead acetate solution was added. A bulky white precipitate indicates the presence of phenols. To test for tannins, 50 mg of the extract was dissolved in 5 ml of distilled water, and to these 2 drops of neutral 5% ferric chloride solution were added. A dark green color indicates the detection of tannins. To test for triterpenoids, the extract (50 mg) was treated with 3 ml chloroform, and then filtered and added with 2 drops of concentrated sulphuric acid, gently shaken and then allowed to stand. The appearance of golden yellow color indicates the presence of triterpenoids. To test for the presence of phytosterols, 2 drops of concentrated sulphuric acid were slowly added to the extract (50 mg) dissolved in 2 ml acetic anhydride. Formation of a brown ring at the junction confirms the presence of phytosterols. For the presence of saponins, 50 mg of the extract was diluted with distilled water to make up to 20 ml in a graduated cylinder and shaken for 15 min. Formation of a 2 cm layer of foam indicates the presence of saponins.

**Table 1:** Phytochemical constituents of *Withania somnifera* roots methanol extract.

Phytochemicals	Test	Methanol extract	
Alkaloids	Wagner’s test	+	
Flavonoids	Bate-Smith and Metcalf test	+	
Glycosides	Borntrager’s test	−	
Phenols	Lead acetate test	+	
Tannins	Braemer’s test	+	
Terpenoids	Salkowski’s test	−	
Phytosterols	Liebermann–Burchard’s test	−	
Saponins	Foam test	+	

‘+’ indicates presence; ‘−’ indicates absence; ‘colors’ indicate the precipitate simulation color for presentness; ‘*’ indicates reactions within 5 min; ‘**’ reactions in 5–20 min; ‘***’ no-reaction up to 24 h.

After the qualitative phytochemical analysis, the determined constituents of the extract that are defined to contribute towards the overall antioxidant activities were quantitated (Table 2) according to standard operating methods^39^, viz*.* using gravimetric analysis for alkaloids, quercetin standard curve (*y* = 0.012*x* + 0.0604; *R*^2^ = 0.9918) for flavonoids, gallic acid curve (*y* = 0.0072*x* + 0.0472; *R*^2^ = 0.9897) for phenols, and tannic acid curve (*y* = 0.0061*x* + 0.0434; *R*^2^ = 0.9895) for tannins. In brief, for quantification of alkaloids, 50 mg of the dried sample was gently mixed with 200 ml of 10% acetic acid in ethanol and allowed to stand for 4 h. The mixture was then concentrated on a water bath for up to one-third of its original volume followed by the addition of ammonium hydroxide to the mixture dropwise until it formed precipitate. After 3 h of the mixture sedimentation, the precipitate was washed with ammonium hydroxide (0.1 M) and then filtered. The filtrate (alkaloids) was dried in an oven and quantitated as a percentage of the dried sample. For the total flavonoid content, 0.3 ml of the extract was mixed with 0.15 ml of sodium nitrite (0.5 M) and 0.15 ml of aluminum chloride hexahydrate (0.3 M) and allowed to stand for 1 h. Then sodium hydroxide was added to the mixture and, after 5 min of mixing, absorbance was recorded at 560 nm. Total flavonoid content was determined using quercetin standard curve as µg quercetin equivalents/mg of the extract. For total phenolic content, 1 ml of the extract was mixed with 9 ml of distilled water. To the mixture, 1 ml of Folin–Ciocalteu’s phenol reagent followed by 10 ml of sodium carbonate solution were added and the total volume was made to 25 ml by adding distilled water and left to react for 90 min. The optical density was determined at 750 nm and the quantity of total phenolic was calculated using the gallic acid curve as µg gallic acid equivalent/mg of the extract. For quantification of tannins, 5 ml of the extract was mixed with 2 ml of ferric chloride in hydrochloric acid (0.1 N) and potassium ferricyanide (0.008 M) and left to react for 10 min. Absorbance was taken at 120 nm and the quantity of tannins was determined using the tannic acid curve as µg tannic equivalent/mg of the extract.

**Table 2:** Basic chemical structures and concentrations of *W. somnifera* roots methanolic extract bioactive constituents that positively influence the overall antioxidant activity of the extract.

Phytochemicals	Chemical structures	Concentrations
Alkaloids		0.27%
Flavonoids		67.57 ± 0.97
Phenols		91.09 ± 0.29
Tannins		35.27 ± 0.51

Concentration of alkaloids expressed as percent of dry weight; Concentrations of flavonoids, phenols, and tannins expressed as mean ± SEM microgram equivalents of respective standards per milligram of extract.

### Animals

This study used a cohort of 1-4-month-old, 48 5xFAD^32^ male mice purchased from the Experimental Animal Center of Tongji Medical College, Huazhong University of Science and Technology, Wuhan, China. All mice were housed under diurnal lighting conditions (12 h darkness/light), at ambient temperature (22 ± 2 °C) and relative humidity (55 ± 5%), and were provided free access to ad libitum food and water. All experimental procedures were conducted in accordance with the internationally accepted principles for laboratory animal use and care as found in the European Community guidelines (EEC Directive of 1986; 86/609/EEC), approved by the Committee for Ethics on Animal Experiments of Tongji Medical College, Huazhong University of Science and Technology (No. S2189). All efforts were made to minimize the number of animals used as well as their suffering, and this study’s reporting of animal experiments complies with the ARRIVE (Animal Research: Reporting of In Vivo Experiments) guidelines.

### Animals grouping and treatment

Male 5xFAD mice (1-month-old, weighing 16.5 ± 2.6 g) were housed under the bioterium conditions described above in groups of 6 animals per ventilated cage from postnatal day 30 (P30) until P130. The mice were divided into 4 groups of 12 in each and were treated with intra-gastric gavage daily for 45 days (P30-75) as Group I, 5xFAD mice treated with 1 ml saline (0.9%)/day, a vehicle control group; Group II, 5xFAD mice treated with 200 mg/kg/day *W. somnifera*, a low dose treatment group; Group III, 5xFAD mice treated with 400 mg/kg/day *W. somnifera*, a high dose treatment group; and Group IV, 5xFAD mice treated with 200 mg/kg/day Resveratrol (Res, ab120726, Abcam), a positive control group. Following cognitional studies, the brains were dissected out at the mice P130 as previously described^32^. And the brain regions associated with AD-related pathology and cognitive decline, namely the frontal cortex, entorhinal cortex, and hippocampus^40,41^, were isolated and used for further studies.

### Cognitional studies

To assess the potential in vivo efficacy of the *W. somnifera* root methanolic extract on the spatial learning and memory of 5xFAD mice, Barnes circular maze and Y-maze spontaneous alternation tasks were used for these procedures require a relatively shorter learning time and involve minimal stress on the mice than the other related paradigms.

#### Barnes circular maze task

The maze acrylic circular platform (92 cm in diameter with 3 mm thickness) was 91 cm elevated above the floor, consisting of 18 circular holes (9.5 cm in diameter) evenly spaced around the periphery. A detachable acrylic black escape box (12 cm × 23 cm × 12 cm) was attached just under one of the hole’s entrance, while the other holes were left open so that the mice cannot enter them. Released from a dark start chamber in the middle of the circular maze after a delay of 10 s each mouse was allowed to explore the maze until it enters the escape box or 5 min elapses for habituation trial, after which the acquisition training phase started in the next 24 h. Following habituation, the mice were trained in spatial learning to escape from the aversive lights by entering the escape box or 3 min elapses. If a mouse fails to enter the escape box within 3 min period, it was gently guided into the escape box and allowed to stay there for 1 min. Training of the mice was carried out in two trials on each day for five consecutive days with an inter-trial interval of approximately 30 min. For each trial, the numbers of error head poke into non-escape holes and the latency time to enter the escape box were recorded to assess performance. Three days after the final session of acquisition training, mice were subjected to a 90 s probe trial to test their spatial learning and memory based on the mice’s time-spent in the virtual target-hole zone, the zone which previously contained an escape box. The mice’s longer time-spent in the target zone was considered to collectively provide a better indicator of spatial learning and memory.

#### Y-maze spontaneous alternation task

The maze consisted of three grey non-reflective plastic arms (5 cm × 30 cm × 12 cm) that were all-placed at 120° from each other with partitions. Following the handling habituation session, each mouse was gently placed into the maze center and allowed to explore the arms for a period of 10 min. The mouse number of arm entries and the subsequent number of triads (entries into three consecutive separate arms) were recorded to determine the percentage of spontaneous alternation. An arm entry was defined as the mouse all-four appendages with its snout oriented towards the end of the arm crosses the threshold of the maze central-zone. The spontaneous alternation behavior was determined as the percentage of the number of triads divided by the number of arm entries minus 2, indicated below in Eq. (1). The mice’s greater percentage of spontaneous alternation was considered to collectively provide a better indicator of spatial learning and working memory.

1 $$ {\text{Spontaneous}}\,{\text{alternation}}\,\% = \frac{{\# \,{\text{triads}}}}{{{\text{Total}}\,{\text{number}}\,{\text{of}}\,{\text{arm}}\,{\text{entries}} - {2}}} \times 100 $$

### Neurochemical studies

#### Enzyme-linked immunosorbent assay (ELISA)

The Aβ_1–42_ level was selectively detected using the Aβ_1–42_ sandwich ELISA kit. To isolate Aβ_1–42_ from the tissue, each mice cerebral cortex and hippocampus were homogenized in ice-cold PBS (0.01 M, pH = 7.4) at 1:9 (tissue weight (g): PBS volume (ml)) and centrifuged at 14,000×*g* for 30 min at 4 °C. The resulting supernatants were used for the assay. The concentration of Aβ_1–42_ in the samples supernatants was determined using the Bradford method^42^, and 100 µl containing 300 µg of proteins from the supernatants fraction were incubated in rabbit anti-mouse Aβ_1–42_ polyclonal antibody-precoated 96-well microplate overnight at 4 °C (KMB3441, Invitrogen). The wells were incubated with anti-rabbit IgG HRP for 30 min at 37 °C, then washed and incubated with stabilized Chromogen for 30 min at 37 °C in the dark. Finally, a stop solution was added to each well, and the plate was read using a microplate reader at 450 nm (Spectramax microplate reader, Molecular Devices).

#### Immunoblotting

Each mice cerebral cortex and hippocampus were homogenized in a buffer containing 50 mM Tris (pH 7.4), 40 mM NaCl, 1 mM EDTA, 0.5% Triton X-100, 1.5 mM Na_3_VO_4_, 50 mM NaF, 10 mM Na_4_P_2_O_7_, 10 mM C_3_H_7_Na_2_O_6_P; added on protease inhibitors cocktail (ab65621, Abcam). The homogenates were transferred into new tubes containing one-third of sample buffer (200 mM Tris–HCl, 8% NaC_12_H_25_SO_4_, and 40% C_3_H_8_O_3_), boiled for 10 min, sonicated, and then cleared by centrifugation at 12,000×*g* for 10 min. The same amount of lysates was separated by 8% SDS-PAGE gel and electroblotted onto nitrocellulose paper (LC2006, ThermoFisher Scientific). Blots were blocked with 5% nonfat dried milk in 0.1% TBS-T (137 mM NaCl, 0.2% Tween 20, and 20 mM Tris/HCl (pH 7.6)) for 1 h. Immunoblots were incubated overnight at 4 °C with a rabbit anti-NCX3 antibody (1:3000) (ab84708, Abcam) diluted in 1% milk in TBS-T and then incubated with a sheep horseradish peroxidase (HRP)-conjugated anti-rabbit (1:10,000) (ab6795, Abcam) in TBS-T for 1 h. Immunoreactive bands were visualized using the enhanced chemiluminescence system (ECL, ThermoFisher). The optical band intensities were determined by densitometric analysis using Image Lab software, and values were normalized to β-actin (loading control).

#### Immunohistochemistry

Mouse brain was cut down into transverse coronal sections at 30 µm using a freezing microtome (SLEE, Mainz, Germany). The sections were permeabilized with 0.5% Triton X-100 in PBS for 30 min and then nonspecific protein binding was blocked with 3% bovine serum albumin (BSA) in PBS for 1 h. Then the sections were incubated with the MOAB-2 primary antibody (mouse IgG2_b_, 1:1000, ab126649, Abcam) overnight at 4 °C. The sections were then washed with PBS (3 × 5 min) and incubated with secondary biotinylated goat anti-mouse at 1:1000 for 1 h. After washing with PBS, sections were incubated with Hoechst 33,342 (10 min) for cell nuclei visualization. Then sections were washed with PBS 3 times, mounted on charged slides, allowed to dry in the dark, and were then incubated with Thioflavin-S staining solution for 10 min. The sections were then dehydrated in a series of alcohols, cleared in xylene, and cover-slipped with 50% glycerin in PBS. Images were acquired using a laser scanning confocal microscope (Zeiss, Tokyo, Japan).

#### Antioxidant activity assay

Each group representative brain extracts were separately homogenized in PBS (50 mM, pH 7.4) at 1:9 (tissue weight (g): PBS volume (ml)) and centrifuged at 7000×*g* for 10 min at 4 °C. Then, the supernatant was used as an enzyme source to measure the superoxide dismutase (SOD) activity and malondialdehyde (MDA) levels. Additionally, to measure the glutathione (GSH) level, the group’s representative brains were homogenized in glutathione reaction buffer and centrifuged at 10,000×*g* for 10 min at 4 °C. The supernatants from the centrifuge were collected and immediately treated with sulfosalicylic acid to precipitate the extra proteins and used as enzyme sources to determine GSH level. The amount of protein was quantified by using bicinchoninic acid (BCA) protein assay kit, and the antioxidant activities were assessed using SOD, MDA, and GSH assay kits. The absorbances were measured shortly after the completion of the reaction using a spectrophotometer at 517 nm.

### Data analysis

Statistical analysis was performed by using Prism Software (GraphPad 8.0 Software, San Diego, CA). All experiments were performed and analyzed in a blinded manner. The data were evaluated as means ± SEM. Statistically significant differences among means were determined by appropriate statistical tests, including repeated-measures, one-way or two-way analysis of variance (ANOVA) followed by Tukey’s multiple comparisons test. Statistical significance was accepted at the 95% confidence level (*p *value < 0.05).

### Ethics approval

Experiments on animals were performed in accordance with the internationally accepted principles for laboratory animal use and care as found in the European Community guidelines (EEC Directive of 1986; 86/609/EEC), approved by the Committee for Ethics on Animal Experiments of Tongji Medical College, Huazhong University of Science and Technology (No. S2189).

## Results

### The current study *W. somnifera* roots satisfied the standards of pharmacognostic quality

Physicochemical composition analysis of the *W. somnifera* roots taken for the current study (loss on drying (2.06%), total ash (4.31%), water soluble ash (1.83%), acid insoluble ash (0.63%), water soluble extractive value (66.62%), and alcohol soluble extractive value (17.34%)), expressed as % of air-dried weight, indicated that the roots satisfy the Ayurvedic Pharmacopoeia of India quality. According to the pharmacognostic standards, the expected values of loss on drying, total ash, water soluble ash, acid insoluble ash, water soluble extractive value, and alcohol soluble extractive value for *W. somnifera* roots are ≤ 8%, ≤ 7%, ≤ 7%, ≤ 1%, ≥ 15%, and ≥ 15%, respectively^34^. In addition, the analytes on the total sugar, total protein, and total lipid of the roots constitution were determined as 7.15%, 4.24%, and 1.41%, respectively.

### *W. somnifera* methanolic extract constitutes antioxidants

Phytochemical screening of *W. somnifera* roots extract (Table 1) confirmed the presence of antioxidants, namely alkaloids, flavonoids, phenols, and tannins. Briefly, Wagner’s test and Lead acetate test for the detections of alkaloids and phenols, respectively, on the *W. somnifera* roots methanolic extract revealed positive results within 5 min per each test reaction. Moreover, screening using the Bate-Smith and Metcalf test for flavonoids and the Braemer’s test for tannins also attested positive results in 15 min and 9 min of each test reaction, respectively. The subsequent quantitative tests on the concentration of the extract phytochemical constitution (Table 2) determined a yield of 0.27% alkaloid per dry weight of the roots, flavonoid content of 67.57 ± 0.97 µg quercetin equivalents/mg, a phenol of 91.09 ± 0.29 µg gallic acid equivalent/mg, and tannins of 35.27 ± 0.51 µg tannic equivalent/mg of extract. These biochemically active constituent organic compounds confer a broad range of *W. somnifera* roots medicinal value.

### *W. somnifera* ameliorates cognition impairment

**Figure 1:**
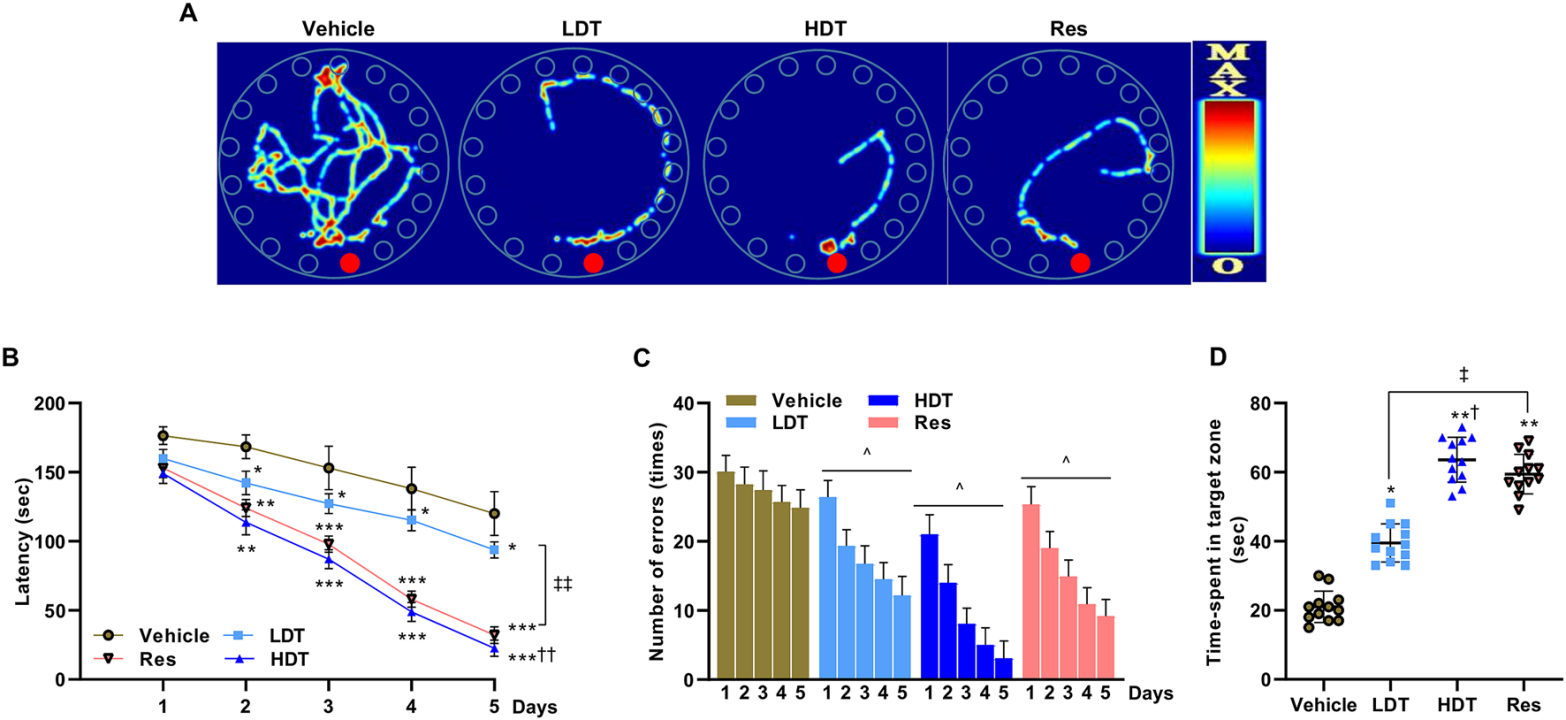
Barnes circular maze task for spatial memory test of 4-month-old 5xFAD mice after 45 consecutive days (P30-75) treatment with *W. somnifera*. Representative heat maps (**A**), and data that compare the latency time (**B**) and the average number of error head pokes (**C**) to enter the escape box during the acquisition training phase. (**D**) The probe trial time-spent in the target zone to locate the hole which previously contained the escape box. The data represent the mean ± SEM. Data were from 12 different mice in each group (n = 12) using repeated-measures ANOVA (in **B**, **C**), two-way ANOVA, one-way ANOVA, Tukey’s multiple comparisons tests. Significance is indicated by *p < 0.05; **p < 0.01; ***p < 0.001 versus vehicle; by ^†^*p* < 0.05, ^††^*p* < 0.01 between *W. somnifera* treated groups; by ^‡^*p* < 0.05, ^‡‡^*p* < 0.01 between *W. somnifera* and Res treated groups; by ^^^*p* < 0.05 between days 1 and 5 (in **C**). *Vehicle* control group, *LDT* low dose treatment, *HDT* high dose treatment, *Res* Resveratrol.

The data obtained after a Barnes circular maze task (Fig. 1) indicate that the mice in the treatment groups showed significant improvements in reaching the escape box quickly and efficiently, whereas, in comparison, the vehicle-treated mice showed much slower learning and more errors. The five consecutive days’ acquisition training demonstrated the vehicle group deficits in spatial learning, consistent with the literature^43,44^, as manifested by prolonged latencies (*p* < 0.05 vs. 200 mg/kg/day *W. somnifera* treatment group, and *p* < 0.001 vs. 400 mg/kg/day *W. somnifera* treatment and 200 mg/kg/day Resveratrol (Res) treatment groups) and increased error rates (*p* < 0.01 vs. 200 mg/kg/day *W. somnifera* treatment group, and *p* < 0.001 vs. 400 mg/kg/day *W. somnifera* treatment and 200 mg/kg/day Res treatment groups) to locate the escape box, while the treatment groups continued the learning (Fig. 1B, C, respectively). Furthermore, analysis of the probe trial data, three days after the final session of acquisition training, provided additional insight on the significant difference between the treatment and vehicle groups in their time spent in the maze virtual target-hole zone, the zone which previously contained an escape box. The result indicated that the treatment groups exhibited a significantly longer time in the target zone compared to the vehicle-treated group (39.5 ± 3.7, 63.6 ± 5.6, and 59.4 ± 6.1 s for the 200 mg/kg/day *W. somnifera*, 400 mg/kg/day *W. somnifera*, and Res treatment groups respectively compared to 21.0 ± 3.4 s for the vehicle group), indicative of spatial memory recovery (Fig. 1D). Of note, there were no statistically significant escape behavior performance differences between the *W. somnifera* higher dose (400 mg/kg/day) and Res treated mice during both the acquisition training and probe trial phases of the current study.

**Figure 2:**
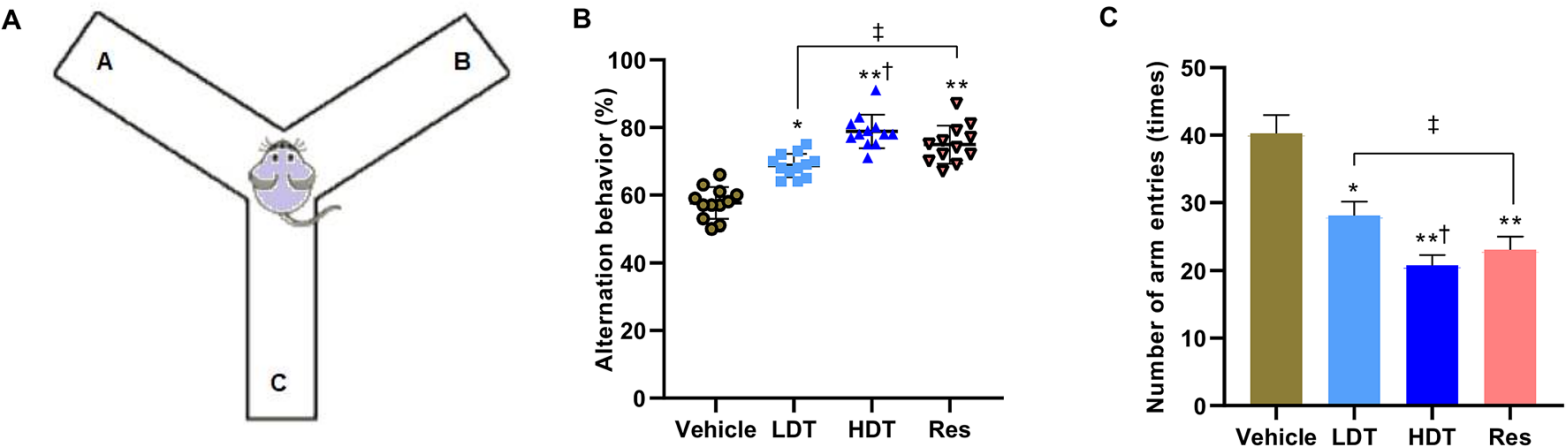
Y-maze spontaneous alternation task for spatial working memory test of 4-month-old 5xFAD mice after 45 consecutive days (P30-75) treatment with *W. somnifera*. Schematic diagram of Y-maze task (**A**), and data that compare the percentage alternation behavior (**B**) and their average number of arm entries (**C**). The data represent the mean ± SEM. Data were from 12 different mice in each group (n = 12) using one-way ANOVA followed by Tukey’s multiple comparisons test. Significance is indicated by *p < 0.05; **p < 0.01 versus vehicle; by ^†^*p* < 0.05 between *W. somnifera* treated groups; by ^‡^*p* < 0.05 between *W. somnifera* and Res treated groups. *Vehicle* control group, *LDT* low dose treatment, *HDT* high dose treatment, *Res* Resveratrol.

Moreover, the same murine data obtained from the Y-maze spontaneous alternation task test (Fig. 2) furtherly substantiated the significant efficacy of the *W. somnifera* roots methanolic extract on spatial learning and working memory of 5xFAD mice. After 45 consecutive days (P30-75) treatment with *W. somnifera* root methanolic extract, the 200 mg/kg/day-treated and 400 mg/kg/day-treated mice showed remarkable improvement in alternation behavior between the arms of the Y-maze task compared to the vehicle-treated 5xFAD mice (ANOVA followed by Tukey’s multiple comparisons test, n = 12 mice/group). Interestingly, there were no statistically significant spontaneous alternation behavior performance differences between the *W. somnifera* higher dose (400 mg/kg/day) and the Res treated 5xFAD mice (average of 79.6 ± 4.2% and 75.4 ± 6.0%, respectively).

### *W. somnifera* blocks the NCX3 suppression induced by Aβ_1–42_ aggregation

**Figure 3:**
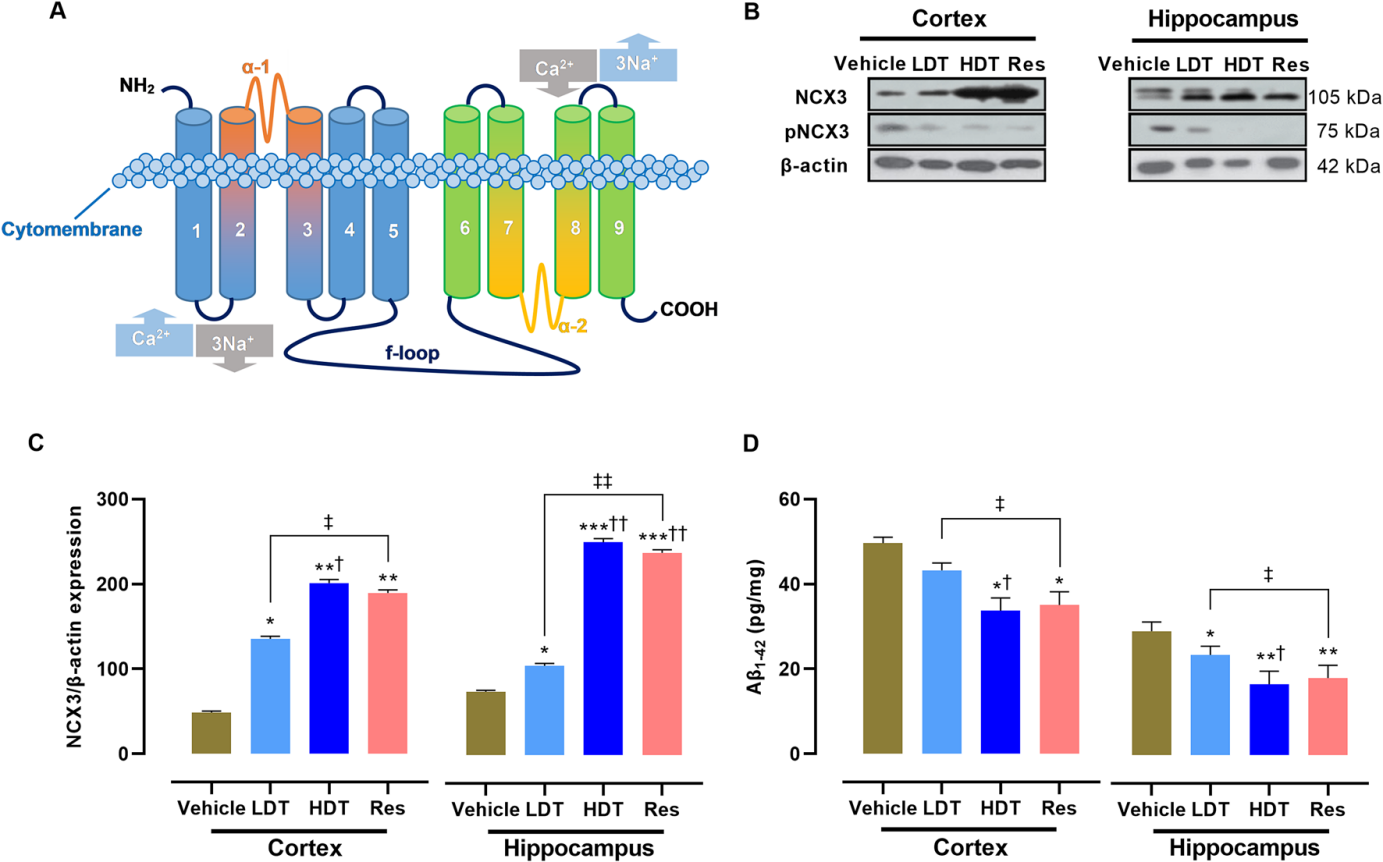
Neurochemical studies for the expression level of NCX3 and Aβ_1–42_ in the cortex and hippocampus homogenates of 4-month-old 5xFAD mice after 45 consecutive days (P30-75) treatment with *W. somnifera*. (**A**) Schematic representation of NCX3 molecular pharmacology. (**B**) NCX3 expression normalized based on a β-actin loading control from the same immunoblot. (**C**) Quantitative analysis of the NCX3 immunoblotting. (**D**) Total Aβ_1–42_ level. The cropped and full-membrane images of immunoblots are presented in Supplementary Figures S2 and S3. The data represent the mean ± SEM. Data were from 4 different mice in each group (n = 4 of two independent sessions) using one-way ANOVA followed by Tukey’s multiple comparisons test. Significance is indicated by *p < 0.05; **p < 0.01; ***p < 0.001 versus vehicle; by ^†^*p* < 0.05, ^††^*p* < 0.01 between *W. somnifera* treated groups; by ^‡^*p* < 0.05, ^‡‡^*p* < 0.01 between *W. somnifera* and Res treated groups. *NCX3* Na^+^–Ca^2+^ exchanger isoform 3, *pNCX3* proteolytic fragment of NCX3, *Vehicle* control group, *LDT* low dose treatment, *HDT* high dose treatment, *Res* Resveratrol.

To examine the effects of *W. somnifera* on the expression level of NCX3 and Aβ_1–42_ in the cortex and hippocampus homogenates of 4-month-old 5xFAD mice brain, immunoblot analysis of the NCX3 expression using a β-actin loading control from the same blot and a sandwich ELISA of the total Aβ_1–42_ were performed. Regarding NCX3 protein expression, significantly decreased band levels on the blot were detected both in the cortex (*p* < 0.01) and the hippocampus (*p* < 0.001) of the vehicle group compared to those of *W. somnifera* higher dose (400 mg/kg/day) and Res treatment groups (Fig. 3B, C). The *W. somnifera* lower dose treatment (200 mg/kg/day) also significantly increased the NCX3 levels both in the cortex (*p* < 0.05) and the hippocampus (*p* < 0.05) compared to that of the vehicle group, as observed in the *W. somnifera* higher dose and Res treatment groups. Noteworthy, a pronounced formation of 75 kDa sized proteolytic fragments were observed in both the cortex and hippocampus of the vehicle group and a few in the hippocampus of the *W. somnifera* lower dose treatment group, but not in the *W. somnifera* higher dose and the Res treatment groups (Fig. 3B). Regarding the level of total Aβ_1–42_ (Fig. 3D), the quantitative analysis by sandwich ELISA showed a significantly reduced expression of Aβ_1–42_ in the cortex and hippocampus of the *W. somnifera* higher dose (*p* < 0.05 and *p* < 0.001, respectively) and Res (*p* < 0.05 and *p* < 0.01, respectively) treatment groups compared to that of the vehicle group. Remarkably, while there is no statistically significant difference in the total Aβ_1–42_ level in the cortex between the *W. somnifera* lower dose treatment and vehicle groups, the overall considerably lower level of total Aβ_1–42_ in the hippocampus than in the cortex of the *W. somnifera* treatment groups is worthy of notice. Furtherly as a point of interest, no significant differences in the cortex and hippocampus levels of NCX3 and Aβ_1–42_ were observed between the *W. somnifera* higher dose and the Res treatment groups. These neurochemical study findings suggest that *W. somnifera* treatment might prevent Aβ_1–42_ deposition and the aggregated Aβ_1–42_-induced NCX3 suppression pathways in the cortex and hippocampus of the 5xFAD mice model.

### *W. somnifera* precludes the Aβ aggregation

**Figure 4:**
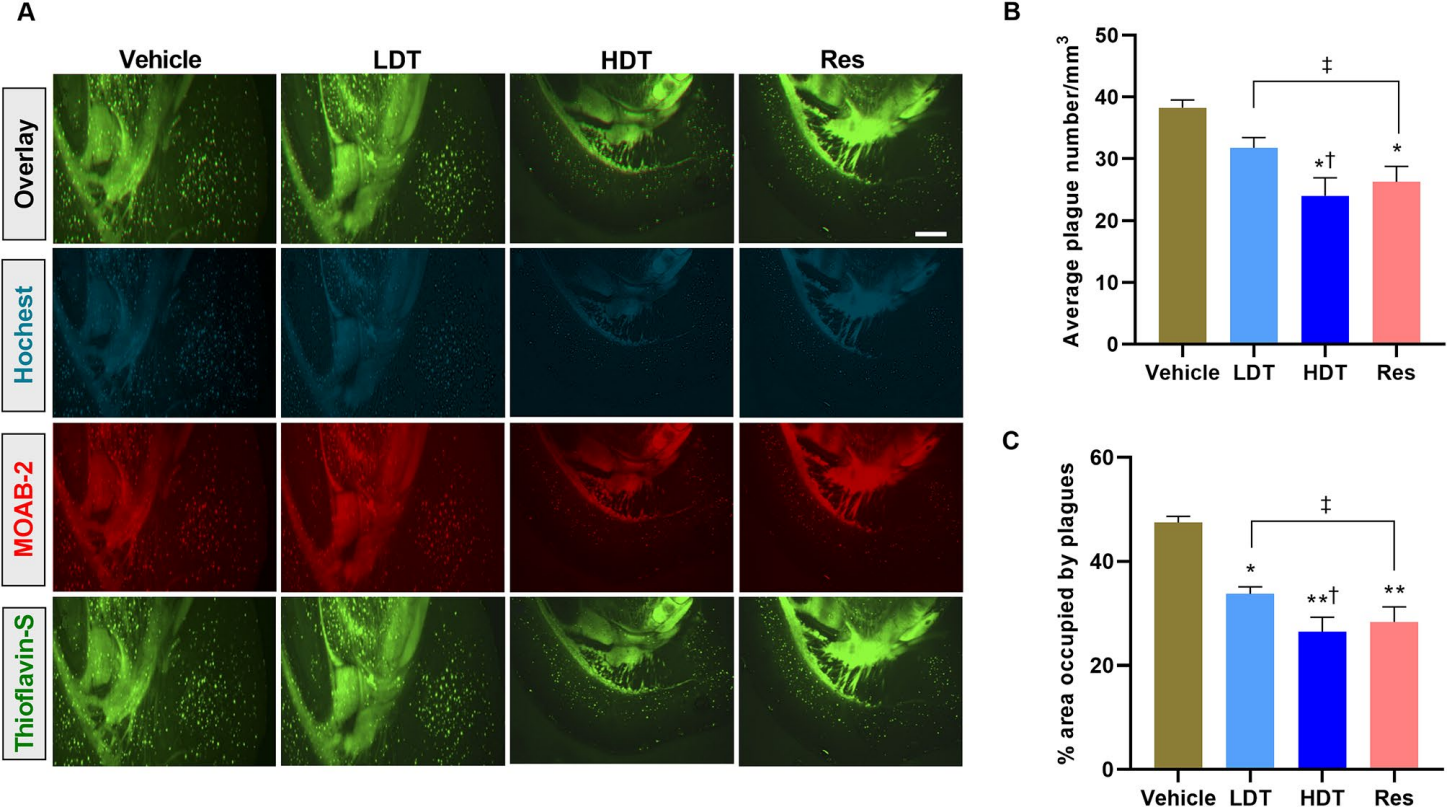
Confocal microscope immunoreactive studies to examine Aβ plaque deposition in the cortex region of 4-month-old 5xFAD mice brain after 45 consecutive days (P30-75) treatment with *W. somnifera*. (**A**) Representative images at × 100 of combined immunofluorescence and Thioflavin-S staining of the mice brain coronal sections, scale bar = 500 μm. (**B**) Quantification of the average number of Aβ plaque deposition. (**C**) Percentage of the area occupied by the plaques. The data represent the mean ± SEM. Data were from 4 different mice in each group (n = 4 of two independent sessions) using one-way ANOVA followed by Tukey’s multiple comparisons test. Significance is indicated by *p < 0.05; **p < 0.01 versus vehicle; by ^†^*p* < 0.05 between *W. somnifera* treated groups; by ^‡^*p* < 0.05 between *W. somnifera* and Res treated groups. *Vehicle* control group, *LDT* low dose treatment, *HDT* high dose treatment, *Res* Resveratrol.

**Figure 5:**
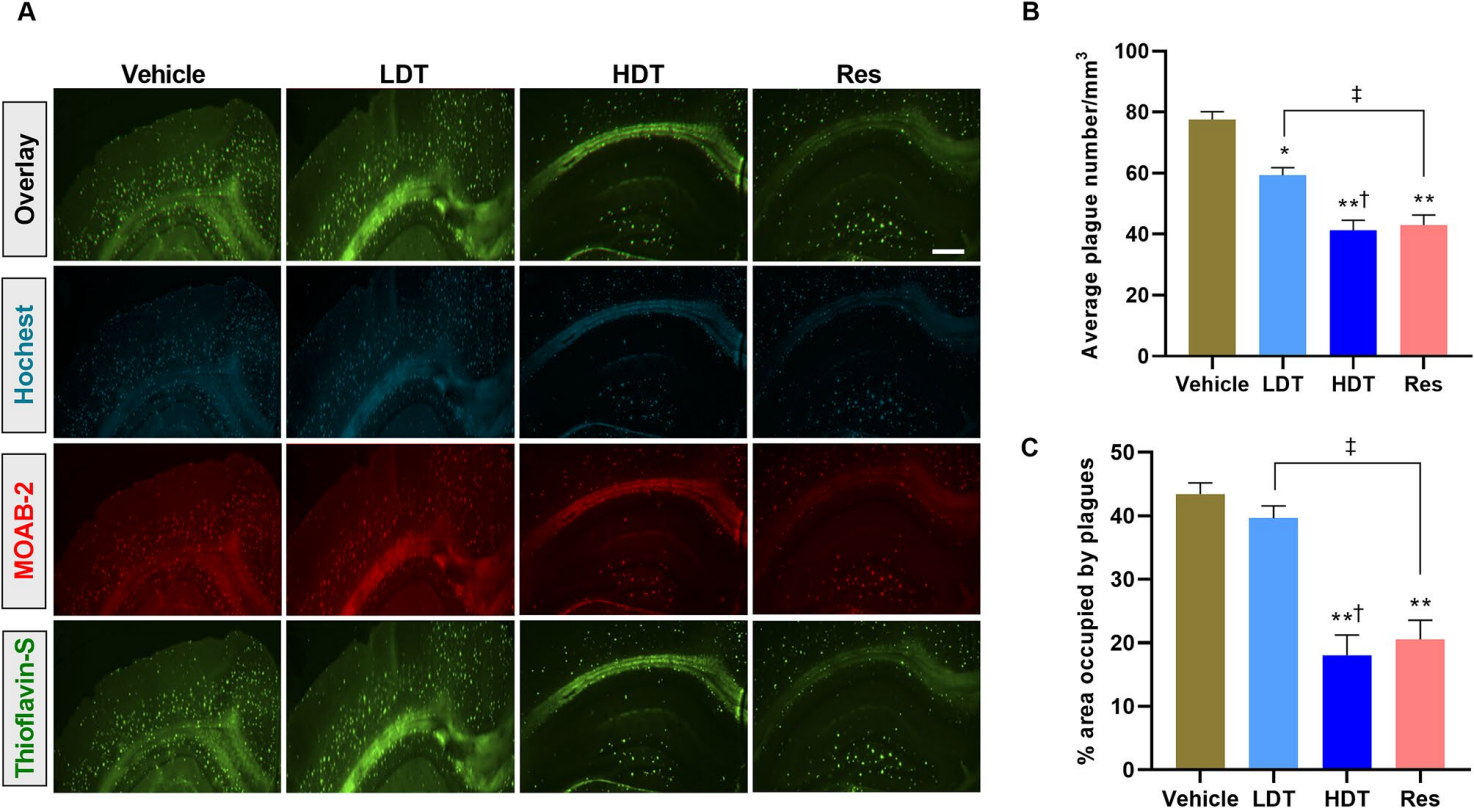
Confocal microscope immunoreactive studies to examine Aβ plaque deposition in the hippocampus region of 4-month-old 5xFAD mice brain after 45 consecutive days (P30-75) treatment with *W. somnifera*. (**A**) Representative images at × 100 of combined immunofluorescence and Thioflavin-S staining of the mice brain coronal sections, scale bar = 500 μm. (**B**) Quantification of the average number of Aβ plaque deposition. (**C**) Percentage of the area occupied by the plaques. The data represent the mean ± SEM. Data were from 4 different mice in each group (n = 4 of two independent sessions) using one-way ANOVA followed by Tukey’s multiple comparisons test. Significance is indicated by *p < 0.05; **p < 0.01 versus vehicle; by ^†^*p* < 0.05 between *W. somnifera* treated groups; by ^‡^*p* < 0.05 between *W. somnifera* and Res treated groups. *Vehicle* control group, *LDT* low dose treatment, *HDT* high dose treatment, *Res* Resveratrol.

To complement the findings obtained from immunoblotting and sandwich ELISA analysis, the effect of the methanolic solution of *W. somnifera* roots extracts on Aβ plaque deposition, a well-documented earlier pathological characteristic of the 5xFAD mice model^45^, was further investigated using a confocal microscope in the 4-month-old 5xFAD mice-brain, cortex and hippocampus tissues. The confocal microscope immunoreactive studies indicated reduced average number of Aβ plaque deposition and percentage of the area occupied by the plaques in both the cortex and hippocampus tissues of the *W. somnifera* and Res treatment groups compared to the vehicle group (Figs. 4, 5, ANOVA followed by Tukey’s multiple comparisons test, mean ± SEM, n = 4 mice/group of two independent sessions). Regarding the cortex (Fig. 4), Thioflavin-S staining for Aβ plaque deposition (Fig. 4A) and quantitative analysis on the average number (Fig. 4B) and percentages of the area occupied by the plaques (Fig. 4C) indicated that the *W. somnifera* higher dose (400 mg/kg/day) and Res treatment groups exhibit notable declines in the number of Aβ plaque deposition (*p* < 0.05, an average of 24.3 ± 3.2 and 26.4 ± 2.7 plaques/mm^3^ for the 400 mg/kg/day *W. somnifera* and Res treatment groups, respectively, while accumulating to an average of 38.5 ± 1.7 plaques/mm^3^) and principally in the distribution of the area occupied by the plaques (*p* < 0.01, 26.5 ± 3.4% and 28.3 ± 3.6% area occupied by the plaques in the 400 mg/kg/day *W. somnifera* and Res treatment groups, respectively, while distributing to 47.4 ± 1.5%) compared to the vehicle group. However, no statistically significant difference was observed in both the average number of plaques/mm^3^ (Fig. 4B) or the percentage of the plaque occupied cortex area (Fig. 4C) between the *W. somnifera* higher dose and Res treatment groups, and in the average number of plaques/mm^3^ between the *W. somnifera* lower dose treatment and the vehicle groups.

As in the cortex of the 4-month-old 5xFAD mice brain, the same groups of murine hippocampus tissue confocal microscope immunoreactive studies exhibited a significant reduction in the average number and distribution of the Aβ plaques/mm^3^ of the 400 mg/kg/day *W. somnifera* and the Res treatment groups compared to the vehicle group (Fig. 5, *p* < 0.01, ANOVA followed by Tukey’s multiple comparisons test, mean ± SEM, n = 4 mice/group of two independent sessions). The reduced production or increased clearance of the Aβ plaque deposition in the hippocampus (Fig. 5B) was significantly higher compared to that of the cortex (Fig. 4B) in the *W. somnifera* and the Res treatment groups (summary of Figs. 4, 5 confocal microscope images can be found as Supplementary Figure S4). These data suggest that the free radical scavenging activity of *W. somnifera*^46,47^ might have contributed to the oral administration of the extract effects in providing protections against Aβ_1–42_ aggregation in the 5xFAD mice cortex and hippocampus. Remarkably, as also observed in the cortex (Fig. 4B, C), no statistically significant difference was observed in the hippocampus tissues on an average number of plaques/mm^3^ (Fig. 5B) and the percentage of the plaque occupied area (Fig. 5C) between the *W. somnifera* higher dose and Res treatment groups.

### *W. somnifera* normalizes oxidative stress

**Figure 6:**
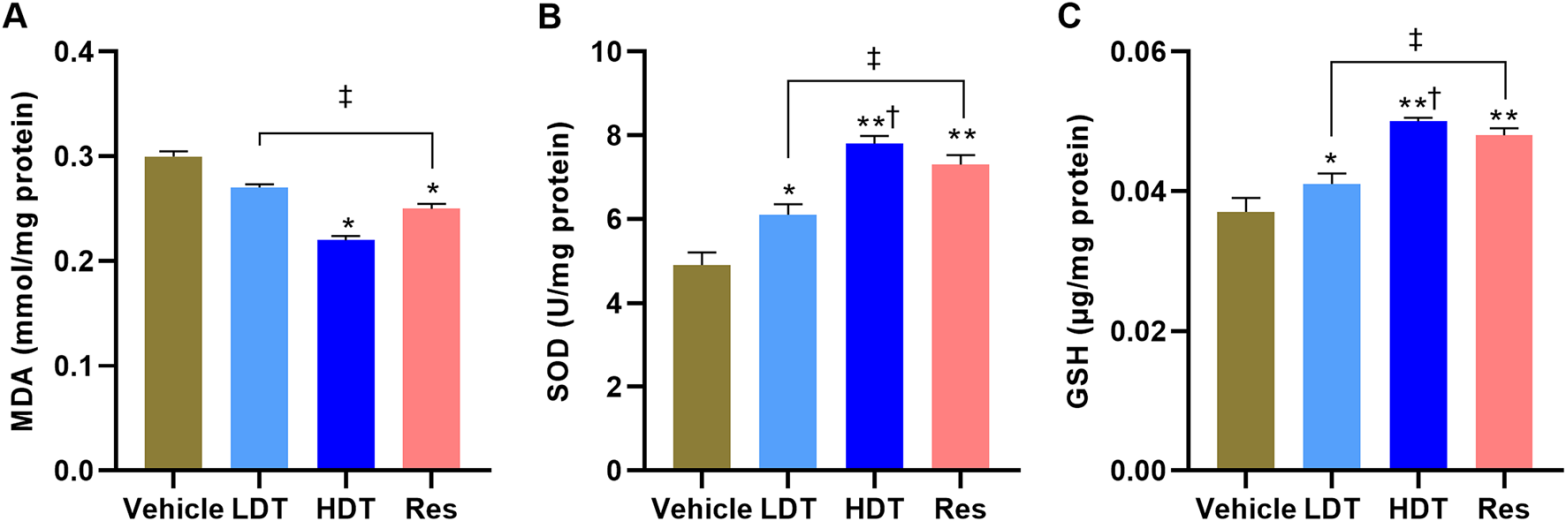
Antioxidant activity assay for the expression level of (**A**) MDA, (**B**) SOD, and (**C**) GSH in the brain tissue homogenates of 4-month-old 5xFAD mice after 45 consecutive days (P30-75) treatment with *W. somnifera*. The data represent the mean ± SEM. Data were from 4 different mice in each group (n = 4 of two independent sessions) using one-way ANOVA followed by Tukey’s multiple comparisons test. Significance is indicated by *p < 0.05 and **p < 0.01 versus vehicle; by ^†^*p* < 0.05 between *W. somnifera* treated groups; by ^‡^*p* < 0.05 between *W. somnifera* and Res treated groups. *MDA* malondialdehyde, *SOD* superoxide dismutase, *GSH* glutathione, *Vehicle* control group, *LDT* low dose treatment, *HDT* high dose treatment, *Res* Resveratrol.

For a further substantiation of the antioxidant effects of *W. somnifera* root-methanolic extract and subsequently to elucidate the extract therapeutic mechanism, oxidative stress markers, including MDA, SOD, and GSH, were measured in the 4-month-old 5xFAD mice brain tissue homogenates (Fig. 6). A one-way ANOVA revealed significant differences in the MDA (*p* < 0.05), as well as both SOD and GSH levels (*p* < 0.01) compared to that of the vehicle group. The marked increase in MDA level in the vehicle group brain was determined reversed with the administration of treatments (*p* < 0.01 verses *W. somnifera* higher dose and the Res-treated mice). And the decreased levels of SOD and GSH in the vehicle group were ameliorated by the interventions (*p* < 0.05 versus *W. somnifera* lower dose; *p* < 0.01 versus *W. somnifera* higher dose and the Res-treated mice). And remarkably, there were no notable differences in the oxidative stress normalization effects of the *W. somnifera* higher dose and Res treatments.

## Discussion

Alzheimer’s disease (AD) is the only cause of global death in the top ten^48^ that cannot be prevented, cured, or even significantly slowed to date. The extracellular aggregation of Aβ_1–42_ protein plaques is regarded as a prominent neuropathological characteristic of AD leading to neurodegeneration and cognitive decline^49–51^. This aggregation of Aβ_1–42_ plaques in the brain is likely caused by aberrant proteolysis of amyloid precursor protein (APP)^51^, and subsequently, the plaques cue calcium-influx-related neuronal damage, including mitochondrial dysfunction, in AD^6,52,53^. For decades, removing the Aβ_1–42_ plaques in the brain has been proposed as the principal determinant to prevent AD^50,51,54^. Indeed, numerous studies using AD transgenic mice with aggregated Aβ_1–42_ plaque evidenced that remotion of these plaques is associated with behavioral improvements^55,56^. In this view, several clinical trials for AD therapeutics have aimed to remove the Aβ_1–42_ plaque from the patient’s brain^57–59^; however, most of these trials have failed^54,60^. These apparent discrepancies suggest that a long time before the formations of the plaques, small forms of Aβ_1–42_ called oligomers appear to be toxic for neurons in such a way that binding of the oligomers to the neurons plasma membrane proteins, such as NCX3, disrupt intraneuronal Ca^2+^ homeostasis in AD. Thus the toxic Aβ_1–42_ oligomers and their accumulation, plaques, altogether appear to be responsible for AD symptoms.

Previous studies of disrupted calcium-influx in AD suggest that aggregation of Aβ_1–42_ plaques in neurons may be responsible for NCX3 downregulation observed in the AD pathological changes^3,6^. However, it is unclear whether the renovation of the aggregated Aβ_1–42_ induced NCX3 downregulation prevents or delays the onset of AD and AD-related pathology. The present study, for the first time, assessed the therapeutic significance of *W. somnifera* roots methanolic extract against AD-related Aβ_1–42_ aggregation, NCX3 suppression, oxidative stress, and cognitional deficits using 5xFAD mice. *W. somnifera* has a long history in the traditional medical system as an ingredient in many formulations prescribed for various aging-related health impairments (e.g., arthritis, stress) to prevent disease and improve overall vitality and longevity^9,61^. However, the therapeutic value of this plant roots extract in neurodegenerative diseases, such as AD, albeit far less well studied. To have a baseline of comparison for the effect of *W. somnifera* in this study, Resveratrol (Res, > 99% purity), a natural polyphenolic compound derived from peanuts, red grapes, blueberries, and other plants^62–64^ was used as a positive control. Res has extensive records for its neuroprotective features, including inhibition of Aβ plaque formation^65–70^ and prevention of cognitive impairment^65,66,70–73^ in AD rodent models. Moreover, due to its antioxidant features^68,70,72^, oral administration of Res is reported to slow aging and increase life span in mice^74–77^. However, to date, there is no specific report on the potential value of Res to enhance the expression level of membrane transport proteins that involve in the mechanisms of cellular Ca^2+^ homeostasis, such as NCX3.

Additionally, an elevated level of Aβ_1–42_ has been attributed to induce oxidative stress that further triggers calcium dyshomeostasis in the AD hippocampus and cortex^78^. Oxidative stress, an increase in production of reactive oxygen species (ROS) and reactive nitrogen species (RNS), such as superoxide radical (O_2_^−^), hydrogen peroxide (H_2_O_2_), hydroxyl radical (HO^−^), nitric oxide (NO), and peroxynitrite (ONOO^−^), causes neuronal dysfunction including cell membrane disintegration and mitochondrial damage leading to AD-pathology^79^. To mitigate oxidative stress in AD, several synthetic antioxidants were formulated for clinical trials over the past decades^80–82^; however, there is no Food and Drug Administration (FDA)-approved antioxidant therapy to date for most of the clinical trials raised significant safety concerns. Consequently, alternative natural antioxidants from plant material extracts, such as Res^72^, have recently received much attention. Of note, using an organic solvent in the extraction medium is documented to maximize yield through optimum solvation of components in the milieu and also to enhance favorable influence in the extract phytochemical constituent’s activity^83,84^. On these bases, herein, methanol was used as a medium for extractions of *W. somnifera* roots constituents. This study methanol extraction yielded a mean of 16.49%, expressed as the mass of extract obtained per 100 g of *W. somnifera* roots, and a yield of 0.27% alkaloid per dry weight of the plant roots, flavonoids content of 67.57 ± 0.97 μg quercetin equivalents/mg, a phenol of 91.09 ± 0.29 μg gallic acid equivalent/mg, and tannins of 35.27 ± 0.51 μg tannic equivalent/mg of the extract (Table 2) which are all consistent with the findings of previously reported studies^84–86^. These phytochemical constituents confirm the antioxidant property^86,87^ of the *W. somnifera* roots methanolic extract as a potent to neutralize free radicals and thereby may represent a preventive role against oxidative stress in AD. Moreover, consistent with the observations of previously reported studies on the acute and sub-acute toxicity effects of standardized extracts of *W. somnifera* roots in rodents^12,14,15^, the current study findings using the daily recorded body weights of the mice (Supplementary Figure S1) during the periods of 200–400 mg/kg/day gavage treatment (P30-75) further ascertains that the *W. somnifera* roots methanolic extract has no potential toxicity. A noteworthy fact is that a rather intravenous administration enhances efficient delivery of extracts bioactive constituents from the plasma into the blood–brain barrier, which correspondingly this mode of treatment alongside the application of advanced purification technique during the extraction process can substantially decrease the effective high dose used in this study.

After a daily for 45 days, from postnatal day 30 (P30) to P75, oral administration of either 200 mg/kg/day or 400 mg/kg/day methanolic solution of *W. somnifera* roots extract as a treatment to rescue cognitive impairment, the 4-month-old 5xFAD mice cognition was assessed using the Barnes circular maze task and Y-maze spontaneous alternation task techniques. Barnes circular maze task was performed to examine whether the *W. somnifera* treated mice display the escape behavior faster than the control mice. The results showed clearly that *W. somnifera* treated mice could find and enter the escape box significantly faster than their cohort vehicle mice (Fig. 1). According to the analysis of the trend across the Barnes maze sessions in latency, the *W. somnifera* treated and the Res treated mice continued learning of escape behavior while comparatively not the vehicle mice. The shorter latencies of escape behavior are presumably a result of improved spatial learning and memory in the treated mice, while the exploring behavior toward the other holes looking for the escape box might have increased the latency in the vehicles. Allied with this notion, studies on the potential role of Theracurmin^43^, Tyrosol^44^, and Metformin^88^ to mitigate AD-related pathology in model mice have adopted a similar protocol.

Reassuringly, consistent with the results obtained from the Barnes circular maze task, the same groups of mice Y-maze spontaneous alternation task data proved the potential in vivo efficacy of the *W. somnifera* roots methanolic extract on the spatial working memory of 5xFAD mice (Fig. 2). Both groups in the daily for 45 days of 200 mg/kg/day and 400 mg/kg/day-treated mice performed better than their cohort vehicle-treated mice on the alternation behavior test. Noteworthy, the alternation behavior of the vehicle-treated 5xFAD mice was consistent with the literature^32^, and there was no statistical difference between the *W. somnifera* higher dose and the Res-treated mice, implying both treatments were sufficient for behavioral improvement. The Y-maze task behavioral test exploits the innate explorative tendency of rodents to evaluate the potential effects of drugs against the underlying pathology of neurodegenerative diseases, such as AD, on spatial learning and working memory^89^.

Subsequent to cognitional studies, the mice brain regions associated with AD-related pathology and cognitive decline, namely frontal cortex, entorhinal cortex, and hippocampus^40,41^, were isolated and used for further neurochemical studies to determine the potential effects of the *W. somnifera* roots methanolic extract in each group. Strikingly, in support of the cognitive assay findings, immunoblotting analysis on the expression level of NCX3 and a sandwich ELISA quantification of total Aβ_1–42_ level in the cortex and hippocampus homogenates, as well as confocal microscope immunoreactive studies on the Aβ plaque deposition in cortex and hippocampus tissues of the undertaken 4-month-old 5xFAD mice, revealed the neuroprotective significance of *W. somnifera* roots methanolic extract to rescue neurodegeneration. Regarding the immunoblotting analysis on the expression level of NCX3 (Fig. 3B, C), the determined results indicate that treatment with *W. somnifera* significantly increases the expression of NCX3 in the cortex and hippocampus. Dysregulation of NCX3, a plasma membrane protein that prominently contributes to the physiological maintenance of Ca^2+^ homeostasis in excitable cells, such as neural, has been reported as an early molecular distortion in AD^90,91^. A recent in vitro documented study^3^, using BHK cells that mimic AD, findings indicate the NCX3 neuroprotective effect as confirmed through decreased cell death in NCX3 stably transfected cells (BHK-NCX3) compared to their wild types (BHK-WT), a further correlation between NCX3 and the neuronal death. The NCX3 molecular pharmacology (Fig. 3A) studies^6,92,93^ demonstrate that this membrane transport protein contains nine-transmembrane segments (TMS), which exists grouped into two based on their locations in the cytomembrane as an extracellular amino-terminal (NH_2_) group, the first five TMS, and the intracellular carboxyl-terminal (COOH) group, the last four TMS. These hydrophobic terminal groups, separated by intracellular hydrophilic loop, named as f-loop, are involved in the transport of Ca^2+^ across the plasma membrane. The NCX3 f.-loop, critical in the terminals activity regulation, contains two alternative splicing sites recognized as the α-1 repeat, between TMS 2 and 3, and the α-2 repeat, between TMS 7 and 8. It is peculiar to underline that the α-1 and α-2-regions of the f-loop determine the molecular sensitivity of NCX3^94^, and therefore, these regions require extra attention in the pharmacological efforts to develop a compound that targets NCX3. Ordinarily, NCX3 migrates as a 105 and a 75 kDa band in an immunoblot assay (Fig. 3B), whereby these band sizes represent the native NCX3 protein and its proteolytic fragment, respectively^3,90^. Noteworthy, the immunoblot for the NCX3 evidenced that *W. somnifera* roots methanolic extract enhances the expression level of NCX3 and remarkably prevents fragmentation of this membrane transport protein (Fig. 3B, C). These results suggest that the *W. somnifera* significance in ameliorating cognitive function may associate with the increased expression level of neuronal membrane NCX3, which is attributable to preventing neuronal cell damage from Ca^2+^ dyshomeostasis. Further relative comparison (Fig. 3C) on the observed robust signal expression (Fig. 3B) of the full-length NCX3 protein levels in the cortex than in the hippocampus of the *W. somnifera* higher dose and the Res treatment groups contrarily revealed a more differential specificity of the treatments benefit to the hippocampal region, suggesting that the effect of the undertaken therapeutic interventions in improving cognitive performance mainly relies on the improved NCX3 regulation in the hippocampus. Additionally, equivalent to the efficacy of the *W. somnifera* higher dose treatment, the investigated Res treatment effects also showed significant roles against NCX3 dysregulation, which suggests sharing of common attributes among these natural products treatment.

Additional imperative findings from the immunoreactive studies (Figs. 3D, 4, 5) are that the oral administration of the *W. somnifera* roots methanolic extract prevented the Aβ_1–42_ aggregation, a well-documented pathological feature of AD^45^, in both the cortex and the hippocampus of 4-month-old 5xFAD mice brain, presumably, through the free radical scavenging activity of the extract phytochemical constituent’s. The phytochemical constituents of *W. somnifera* roots methanol extract (Table 1) include alkaloids, flavonoids, and phenols, which are factual antioxidants^95^. Although the underlying mechanisms of the potent effect of *W. somnifera* roots extract in Aβ metabolism remain unclear, findings of a reported study using AD transgenic mice also shed light on the extract’s ability to enhance efflux of Aβ from the brain to the plasma and eventually lead to its degradation in the periphery^96^. The observed reduction in the Aβ_1–42_ deposition and formation of NCX3 proteolytic fragments in the cortex and hippocampus of the treated 5xFAD mice model compared to their cohort vehicle group suggest that *W. somnifera* treatment might prevent intraneuronal Aβ_1–42_ aggregation and the aggregated Aβ_1–42_-induced NCX3 suppression pathways in the AD pathology.

In line with the extract phytochemical screening findings, as expected, oxidative markers, namely MDA, SOD, and GSH levels, were normalized by *W. somnifera* treatments as evidenced using antioxidant activity assay (Fig. 6). The assay analysis demonstrates that the increased MDA level and decreased SOD and GSH levels in the vehicle-treated 4-month-old 5xFAD mice were significantly reversed by the daily for 45-days (P30-75) administration of 400 mg/kg/day methanolic solution of *W. somnifera* roots extract, suggesting the pharmaceutical significance of this extract constituents to protect neuronal cells from oxidative damage. Antioxidants have been proposed to neutralize the elevated level of Aβ_1–42_-induced free radicals that causes oxidative stress and Ca^2+^ dyshomeostasis in the AD pathology^97,98^; however, formulation of an antioxidant therapy that satisfies the safety concerns requirement remains to be determined. Notably, alongside its phytochemical constituent’s antioxidant feature, the herein observed substantial margin of decreased Aβ protein plaque deposition in the treated mice brain regions associated with AD-related pathology compared to that of vehicles further unveils the pharmaceutical significance of *W. somnifera* roots against AD neurodegeneration.

Prevailing basic science translational researches involving various models^20,99–102^ and clinical trials^103,104^, on the *W. somnifera* extracts pharmacological value in improving memory and cognitive function, enlighten several unmet-gaps that require attention for further rigorous investigations to suitably lead to meaningful clinical outcomes, including objectively characterizing the pharmacologically vital constituents of the extract, distinctively determining the molecular mechanisms underlying contributions in the efficacy of the undertaken extract, and using defined and optimized extract constituents to formulate standardized therapeutics strategy for AD. Studies that evaluated the therapeutic remedial activity of the *W. somnifera* extract, using in vitro and in vivo neurodegeneration model systems, indicate that the extract mechanisms of effects include attenuation of neuroinflammation and increases in the level of autophagic markers in the brain^20,100^, and augmentation of cortical acetylcholine esterase, synaptic plasticity, and neuronal cell survival^101,102^. Further extended clinical trials also highlight the efficacy of *W. somnifera* extract in moderating age-related memory, information processing, attention and executive function impairments^103,104^, and other clinical benefits that can profoundly impact mental wellbeing, including reducing mental stress and anxiety^105,106^. In considering its Ayurvedic medicinal value, at the moment there is a noteworthy overwhelming computational^107,108^, basic science^109,110^, and clinical research^111,112^ momentum and excitement to evaluate the *W. somnifera* extract in chemoprophylaxis against COVID-19 disease^113^ and the long-term effect of COVID-19, post-COVID condition, collateral damage potential contributions in increasing the COVID-19-patients risk of future neurodegenerative diseases, such as AD, where increasing age is a common risk factor in both of these severe conditions, although the effects are not yet explicit. In all, as the investigations looking for an efficient novel therapeutic agent from the plant standardized extracts or bioactive constituents continue, the available research findings strongly suggest considerations to introduce *W. somnifera* roots in nutritional supplementation regimen formulations as an adjunctive treatment for patients with Alzheimer’s neurodegeneration.

In this study context, further studies using NCX3 knockout models would allow for a better understanding of the therapeutic role of *W. somnifera* against aberrant Ca^2+^ influx-induced neurodegeneration, such as AD, as negative controls to uncover novel potential underlying molecular mechanisms of the extract effects in the NCX3 regulation under physiological conditions and during pathologic brain function. Moreover, the chances of observing enhanced progressive improvement in the study mice’s cognitive function for the effects of the *W. somnifera* therapeutic intervention on NCX3 is limited by both the sensitivity and the range of the behavioral tasks yet available. Overall, for the first time, this study reports that *W. somnifera* ameliorates AD-related pathology, including downregulated NCX3 and cognitive impairment in the 5xFAD mouse model, establishing *W. somnifera* as a promising candidate to benefit a model with brain integrity challenged by forced amyloidosis created via expression of a mutant human APP transgene.

## Conclusions

In summary, the proposed mechanisms of the *W. somnifera* roots methanolic extract neuroprotective effects against aggregated Aβ_1–42_-induced NCX3 suppression in the 5xFAD mouse model include enhancement of antioxidative activities, a decrease in the Aβ_1–42_ aggregation, rectification of NCX3 expression, and likely thereby protection against Ca^2+^ dyshomeostasis induced neuronal cell death. These effects of the extract could attribute to the multiple antioxidant phytochemical constituents, and as such, which are likely to have several mechanisms of action. Thus, the *W. somnifera* roots methanolic extract, with no indications of toxicity, might be regarded for the development of a potential drug candidate in the therapeutics approach or prevention of AD.

## Supplementary Information

Supplementary Figures.

## Data Availability

The data and materials that support the findings of this study are available on request under-identification policy to the corresponding authors.

## Abbreviations

NCX: Na^+^–Ca^2^^+^ exchanger
NCX3: Na^+^–Ca^2^^+^ exchanger isoform 3
pNCX3: Proteolytic fragment of NCX3
AD: Alzheimer’s disease
5xFAD: 5 Familial AD mutations
Aβ: Amyloid-β
APP: Amyloid precursor protein
PSEN: Presenilin
LDT: Low dose treatment
HDT: High dose treatment
Res: Resveratrol
ELISA: Enzyme-linked immunosorbent assay
MOAB-2: Monoclonal Antibody-2
MDA: Malondialdehyde
SOD: Superoxide dismutase
GSH: Glutathione

## References

[1] Iwamoto T (2004). Forefront of Na^+^/Ca^2+^ exchanger studies: Molecular pharmacology of Na^+^/Ca^2+^ exchange inhibitors. J. Pharmacol. Sci..

[2] Verkhratsky A, Trebak M, Perocchi F, Khananshvili D, Sekler I (2018). Crosslink between calcium and sodium signalling. Exp. Physiol..

[3] Afewerky HK, Li H, Pei P, Zhang TM, Lu YM (2019). Contribution of sodium–calcium exchanger isoform-3 in Aβ_1–42_ induced cell death. Neuropsychiatry (London).

[4] Polumuri SK, Ruknudin A, McCarthy MM, Perrot-Sinal TS, Schulze DH (2002). Sodium–calcium exchanger NCX1, NCX2, and NCX3 transcripts in developing rat brain. Ann. N. Y. Acad. Sci..

[5] Linck B, Qiu Z, Hilgemann DW, Philipson KD (1997). Functional comparison of three different isoforms of the sodium–calcium exchanger (NCX1, NCX2, NCX3). Biophys. J..

[6] Afewerky HK, Li H, Zhang T, Lu Y (2016). Roles of sodium–calcium exchanger isoform-3 toward calcium ion regulation in Alzheimer’s disease. Alzheimer’s Dis. Parkinsonism.

[7] Hilge M (2012). Ca^2+^ regulation of ion transport in the Na^+^/Ca^2+^ exchanger. J. Biol. Chem..

[8] Mirjalili MH, Moyano E, Bonfill M, Cusido RM, Palazon J (2009). Steroidal lactones from *Withania somnifera*, an ancient plant for novel medicine. Molecules.

[9] Afewerky HK (2021). Critical review of the *Withania somnifera* (L.) Dunal: Ethnobotany, pharmacological efficacy, and commercialization significance in Africa. Bull. Natl. Res. Cent..

[10] Mukherjee PK (2021). *Withania somnifera* (L.) Dunal—Modern perspectives of an ancient Rasayana from Ayurveda. J. Ethnopharmacol..

[11] Ven Murthy MR, Ranjekar PK, Ramassamy C, Deshpande M (2010). Scientific basis for the use of Indian ayurvedic medicinal plants in the treatment of neurodegenerative disorders: Ashwagandha. Cent. Nerv. Syst. Agents Med. Chem..

[12] Prabu PC, Panchapakesan S, Raj CD (2013). Acute and sub-acute oral toxicity assessment of the hydroalcoholic extract of *Withania somnifera* roots in Wistar rats. Phytother. Res..

[13] Sharma S, Dahanukar S, Karandikar SM (1984). Effects of long-term administration of the roots of ashwagandha and shatavari in rats. Indian Drugs.

[14] Prabu PC, Panchapakesan S (2015). Prenatal developmental toxicity evaluation of *Withania somnifera* root extract in Wistar rats. Drug Chem. Toxicol..

[15] Sharada AC, Solomon FE, Devi PU (2008). Toxicity of *Withania somnifera* root extract in rats and mice. Int. J. Pharmacogn..

[16] Seenivasagam R, Sathiyamoorthy S, Hemavathi K (2011). Therapeutic impacts of Indian and Korean ginseng on human beings. Int. J. Immunol. Stud..

[17] Shah N (2015). Combinations of Ashwagandha leaf extracts protect brain-derived cells against oxidative stress and induce differentiation. PLoS ONE.

[18] Henley AB (2017). *Withania somnifera* root extract enhances chemotherapy through 'priming'. PLoS ONE.

[19] Saykally JN (2017). *Withania somnifera* extract protects model neurons from *in vitro* traumatic injury. Cell Transplant..

[20] Dutta K, Patel P, Julien JP (2018). Protective effects of *Withania somnifera* extract in SOD1(G93A) mouse model of amyotrophic lateral sclerosis. Exp. Neurol..

[21] Kurapati KR, Atluri VS, Samikkannu T, Nair MP (2013). Ashwagandha (*Withania somnifera*) reverses beta-amyloid1-42 induced toxicity in human neuronal cells: Implications in HIV-associated neurocognitive disorders (HAND). PLoS ONE.

[22] Kuboyama T, Tohda C, Komatsu K (2006). Withanoside IV and its active metabolite, sominone, attenuate Abeta(25–35)-induced neurodegeneration. Eur. J. Neurosci..

[23] Bhattarai JP, Park SJ, Han SK (2013). Potentiation of NMDA receptors by *Withania somnifera* on hippocampal CA1 pyramidal neurons. Am. J. Chin. Med..

[24] Tiwari S (2018). Withaferin A suppresses beta Amyloid in APP expressing cells: Studies for Tat and Cocaine associated neurological dysfunctions. Front. Aging Neurosci..

[25] Dar NJ (2017). Withanone, an active constituent from *Withania somnifera*, affords protection against NMDA-induced excitotoxicity in neuron-Like cells. Mol. Neurobiol..

[26] Kumar G, Patnaik R (2016). Exploring neuroprotective potential of *Withania somnifera* phytochemicals by inhibition of GluN2B-containing NMDA receptors: An in silico study. Med. Hypotheses.

[27] Alzheimer's Association (2020). Alzheimer's disease facts and figures. Alzheimer's Dement.

[28] Mullan M (1992). A pathogenic mutation for probable Alzheimer's disease in the APP gene at the N-terminus of beta-amyloid. Nat. Genet..

[29] Eckman CB (1997). A new pathogenic mutation in the APP gene (I716V) increases the relative proportion of A beta 42(43). Hum. Mol. Genet..

[30] Goate A (1991). Segregation of a missense mutation in the amyloid precursor protein gene with familial Alzheimer's disease. Nature.

[31] Citron M (1998). Additive effects of PS1 and APP mutations on secretion of the 42-residue amyloid beta-protein. Neurobiol. Dis..

[32] Oakley H (2006). Intraneuronal beta-amyloid aggregates, neurodegeneration, and neuron loss in transgenic mice with five familial Alzheimer's disease mutations: Potential factors in amyloid plaque formation. J. Neurosci..

[33] World Flora Online (WFO). http://www.worldfloraonline.org/. Accessed 21 Dec 2018.

[34] Anonymous, The Ayurvedic Pharmacopoeia of India, Vol. 1, 1st English ed., (Department of Health, Ministry of Health and Family Welfare, Government of India, 1989).

[35] Dutta S, Dey P, Chaudhuri TK (2013). Quantification and correlation of the bioactive phytochemicals of *Croton bonplandianum* leaves of sub-Himalayan region of West Bengal *Asian J Pharma*. Clin Res.

[36] Harborne JB (1984). Phytochemical Methods—A Guide to Modern Techniques of Plant Analysis.

[37] Sofowora A (1993). Recent trends in research into African medicinal plants. J. Ethnopharmacol..

[38] Trease GE, Evans WC (1996). Pharmacognosy.

[39] Shabbir M, Khan MR, Saeed N (2013). Assessment of phytochemicals, antioxidant, anti-lipid peroxidation and anti-hemolytic activity of extract and various fractions of *Maytenus royleanus* leaves. BMC Complement. Altern. Med..

[40] Kaup AR, Mirzakhanian H, Jeste DV, Eyler LT (2011). A review of the brain structure correlates of successful cognitive aging. J. Neuropsychiatry Clin. Neurosci..

[41] Wu A (2019). Association of brain magnetic resonance imaging signs with cognitive outcomes in persons with nonimpaired cognition and mild cognitive impairment. JAMA Netw Open.

[42] Bradford MM (1976). A rapid and sensitive method for the quantitation of microgram quantities of protein utilizing the principle of protein-dye binding. Anal. Biochem..

[43] Kim J (2019). Theracurmin ameliorates cognitive dysfunctions in 5xFAD mice by improving synaptic function and mitigating oxidative stress. Biomol. Ther. (Seoul).

[44] Taniguchi K (2019). Tyrosol reduces amyloid-beta oligomer neurotoxicity and alleviates synaptic, oxidative, and cognitive disturbances in Alzheimer's disease model mice. J. Alzheimers Dis..

[45] Deba F, Peterson S, Hamouda AK (2019). An animal model to test reversal of cognitive decline associated with beta-amyloid pathologies. Methods Mol. Biol..

[46] Senthil K, Thirugnanasambantham P, Oh TJ, Kim SH, Choi HK (2015). Free radical scavenging activity and comparative metabolic profiling of *in vitro* cultured and field grown *Withania somnifera* roots. PLoS ONE.

[47] Sumathi S, Padma PR, Gathampari S, Vidhya S (2007). Free radical scavenging activity of different parts of *Withania somnifera*. Anc. Sci. Life.

[48] World Health Organization. *Global Health Workforce Statistics* (December 2018 Update) (World Health Organization Dashboards, 2018).

[49] Barage SH, Sonawane KD (2015). Amyloid cascade hypothesis: Pathogenesis and therapeutic strategies in Alzheimer's disease. Neuropeptides.

[50] Edwards FA (2019). A unifying hypothesis for Alzheimer's disease: From plaques to neurodegeneration. Trends Neurosci..

[51] Selkoe DJ, Hardy J (2016). The amyloid hypothesis of Alzheimer's disease at 25 years. EMBO Mol. Med..

[52] Green KN (2009). Calcium in the initiation, progression and as an effector of Alzheimer's disease pathology. J. Cell. Mol. Med..

[53] Reddy PH, Beal MF (2008). Amyloid beta, mitochondrial dysfunction and synaptic damage: Implications for cognitive decline in aging and Alzheimer's disease. Trends Mol. Med..

[54] Huang YM, Shen J, Zhao HL (2019). Major clinical trials failed the amyloid hypothesis of Alzheimer's disease. J. Am. Geriatr. Soc..

[55] Janus C (2000). A beta peptide immunization reduces behavioural impairment and plaques in a model of Alzheimer's disease. Nature.

[56] Morgan D (2000). A beta peptide vaccination prevents memory loss in an animal model of Alzheimer's disease. Nature.

[57] Makin S (2018). The amyloid hypothesis on trial. Nature.

[58] Pagnier GJ (2018). Novel botanical drug DA-9803 prevents deficits in Alzheimer's mouse models. Alzheimers Res. Ther..

[59] Sevigny J (2016). The antibody aducanumab reduces Abeta plaques in Alzheimer's disease. Nature.

[60] Panza F, Lozupone M, Logroscino G, Imbimbo BP (2019). A critical appraisal of amyloid-beta-targeting therapies for Alzheimer disease. Nat. Rev. Neurol..

[61] Pradhan R (2017). Longevity and healthy ageing genes FOXO3A and SIRT3: Serum protein marker and new road map to burst oxidative stress by *Withania somnifera*. Exp. Gerontol..

[62] Neves AR, Lucio M, Lima JLC, Reis S (2012). Resveratrol in medicinal chemistry: A critical review of its pharmacokinetics, drug-delivery, and membrane interactions. Curr. Med. Chem..

[63] Piotrowska H, Kucinska M, Murias M (2012). Biological activity of piceatannol: Leaving the shadow of resveratrol. Mutat. Res..

[64] Smoliga JM, Baur JA, Hausenblas HA (2011). Resveratrol and health—A comprehensive review of human clinical trials. Mol. Nutr. Food Res..

[65] Braidy N (2016). Resveratrol as a potential therapeutic candidate for the treatment and management of Alzheimer's disease. Curr. Top. Med. Chem..

[66] Chen Y (2019). Resveratrol improves cognition and decreases amyloid plaque formation in Tg6799 mice. Mol. Med. Rep..

[67] Dhakal S (2019). Dietary polyphenols: A multifactorial strategy to target Alzheimer's disease. Int. J. Mol. Sci..

[68] Feng XW (2013). Resveratrol inhibits beta-amyloid-induced neuronal apoptosis through regulation of SIRT1-ROCK1 signaling pathway. PLoS ONE.

[69] Porquet D (2014). Neuroprotective role of trans-resveratrol in a murine model of familial Alzheimer's disease. J. Alzheimers Dis..

[70] Wang R, Zhang Y, Li J, Zhang C (2017). Resveratrol ameliorates spatial learning memory impairment induced by Abeta1-42 in rats. Neuroscience.

[71] Gocmez SS (2016). Protective effects of resveratrol on aging-induced cognitive impairment in rats. Neurobiol. Learn. Mem..

[72] Gomes BAQ (2018). Neuroprotective mechanisms of resveratrol in Alzheimer's disease: Role of SIRT1. Oxid. Med. Cell. Longev..

[73] Lange KW, Li SM (2018). Resveratrol, pterostilbene, and dementia. BioFactors.

[74] Baur JA (2006). Resveratrol improves health and survival of mice on a high-calorie diet. Nature.

[75] Holme AL, Pervaiz S (2007). Resveratrol in cell fate decisions. J. Bioenerg. Biomembr..

[76] Orallo F (2008). Trans-resveratrol: A magical elixir of eternal youth?. Curr. Med. Chem..

[77] Valenzano DR, Cellerino A (2006). Resveratrol and the pharmacology of aging: A new vertebrate model to validate an old molecule. Cell Cycle.

[78] Padurariu M (2013). The oxidative stress hypothesis in Alzheimer's disease. Psychiatr. Danub..

[79] Reddy PH (2012). Abnormal mitochondrial dynamics and synaptic degeneration as early events in Alzheimer's disease: Implications to mitochondria-targeted antioxidant therapeutics. Biochim. Biophys. Acta.

[80] Arlt S, Muller-Thomsen T, Beisiegel U, Kontush A (2012). Effect of one-year vitamin C- and E-supplementation on cerebrospinal fluid oxidation parameters and clinical course in Alzheimer's disease. Neurochem. Res..

[81] Galasko DR (2012). Antioxidants for Alzheimer disease: A randomized clinical trial with cerebrospinal fluid biomarker measures. Arch. Neurol..

[82] Miller ER (2005). Meta-analysis: High-dosage vitamin E supplementation may increase all-cause mortality. Ann. Intern. Med..

[83] Eloff JN (1998). Which extractant should be used for the screening and isolation of antimicrobial components from plants?. J. Ethnopharmacol..

[84] Ganguly B, Kumar N, Ahmad AH, Rastogi SK (2018). Influence of phytochemical composition on in vitro antioxidant and reducing activities of Indian ginseng [*Withania somnifera* (L.) Dunal] root extracts. J. Ginseng Res..

[85] Hameed A, Akhtar N (2018). Comparative chemical investigation and evaluation of antioxidant and tyrosinase inhibitory effects of *Withania somnifera* (L.) Dunal and *Solanum nigrum* (L.) Berries. Acta. Pharm..

[86] Pisoschi AM, Pop A, Cimpeanu C, Predoi G (2016). Antioxidant capacity determination in plants and plant-derived products: A review. Oxid. Med. Cell. Longev..

[87] Shivakumar A, Yogendra Kumar MS (2018). Critical review on the analytical mechanistic steps in the evaluation of antioxidant activity. Crit. Rev. Anal. Chem..

[88] Farr SA (2019). Metformin improves learning and memory in the SAMP8 mouse model of Alzheimer's disease. J. Alzheimers Dis..

[89] Liu T, Bai W, Xia M, Tian X (2018). Directional hippocampal-prefrontal interactions during working memory. Behav. Brain Res..

[90] Moriguchi S (2018). Reduced expression of Na^+^/Ca^2+^ exchangers is associated with cognitive deficits seen in Alzheimer's disease model mice. Neuropharmacology.

[91] Moriguchi S, Kita S, Iwamoto T, Fukunaga K (2018). Dysfunction of Na^+^/Ca^2+^ exchangers is associated with cognitive decline in Alzheimer's disease. Nihon Yakurigaku Zasshi.

[92] Molinaro P (2020). Genetically modified mice to unravel physiological and pathophysiological roles played by NCX isoforms. Cell Calcium.

[93] Sisalli MJ (2020). Nuclear-encoded NCX3 and AKAP121: Two novel modulators of mitochondrial calcium efflux in normoxic and hypoxic neurons. Cell Calcium.

[94] Annunziato L, Secondo A, Pignataro G, Scorziello A, Molinaro P (2020). New perspectives for selective NCX activators in neurodegenerative diseases. Cell Calcium.

[95] Dar NJ, Hamid A, Ahmad M (2015). Pharmacologic overview of *Withania somnifera*, the Indian ginseng. Cell. Mol. Life Sci..

[96] Sehgal N (2012). *Withania somnifera* reverses Alzheimer's disease pathology by enhancing low-density lipoprotein receptor-related protein in liver. Proc. Natl. Acad. Sci. USA.

[97] Pennisi M (2017). Inflammasomes, hormesis, and antioxidants in neuroinflammation: Role of NRLP3 in Alzheimer disease. J. Neurosci. Res..

[98] Williams DM, Hagg S, Pedersen NL (2019). Circulating antioxidants and Alzheimer disease prevention: A Mendelian randomization study. Am. J. Clin. Nutr..

[99] Alzoubi KH (2019). *Withania somnifera* root powder protects againist post-traumatic stress disorder-induced memory impairment. Mol. Biol. Rep..

[100] Kumar S, Phaneuf D, Julien JP (2021). Withaferin-A treatment alleviates TAR DNA-binding protein-43 pathology and improves cognitive function in a mouse model of FTLD. Neurotherapeutics.

[101] Gupta M, Kaur G (2019). *Withania somnifera* (L.) Dunal ameliorates neurodegeneration and cognitive impairments associated with systemic inflammation. BMC Complement. Altern. Med..

[102] Yadav CS (2010). Propoxur-induced acetylcholine esterase inhibition and impairment of cognitive function: Attenuation by *Withania somnifera*. Indian J. Biochem. Biophys..

[103] Choudhary D, Bhattacharyya S, Bose S (2017). Efficacy and safety of Ashwagandha (*Withania somnifera* (L.) Dunal) root extract in improving memory and cognitive functions. J. Diet. Suppl..

[104] Chengappa KN (2013). Randomized placebo-controlled adjunctive study of an extract of *Withania somnifera* for cognitive dysfunction in bipolar disorder. J. Clin. Psychiatry.

[105] Langade D, Kanchi S, Salve J, Debnath K, Ambegaokar D (2019). Efficacy and safety of Ashwagandha (*Withania somnifera*) root extract in insomnia and anxiety: A double-blind, randomized, placebo-controlled study. Cureus.

[106] Lopresti AL, Smith SJ, Malvi H, Kodgule R (2019). An investigation into the stress-relieving and pharmacological actions of an Ashwagandha (*Withania somnifera*) extract: A randomized, double-blind, placebo-controlled study. Medicine (Baltimore).

[107] Tripathi MK (2021). Identification of bioactive molecule from *Withania somnifera* (Ashwagandha) as SARS-CoV-2 main protease inhibitor. J. Biomol. Struct. Dyn..

[108] Srivastava A (2020). Exploring nature's bounty: Identification of *Withania somnifera* as a promising source of therapeutic agents against COVID-19 by virtual screening and in silico evaluation. J. Biomol. Struct. Dyn..

[109] Balkrishna A (2021). Withanone from *Withania somnifera* attenuates SARS-CoV-2 RBD and host ACE2 interactions to rescue spike protein induced pathologies in humanized Zebrafish model. Drug Des. Dev. Ther..

[110] Balkrishna A, Haldar S, Singh H, Roy P, Varshney A (2021). Coronil, a tri-herbal formulation, attenuates spike-protein-mediated SARS-CoV-2 viral entry into human alveolar epithelial cells and pro-inflammatory cytokines production by inhibiting spike protein-ACE-2 interaction. J. Inflamm. Res..

[111] Chopra A, Srikanth N, Patwardhan B, A. C. R. Group (2021). *Withania somnifera* as a safer option to hydroxychloroquine in the chemoprophylaxis of COVID-19: Results of interim analysis. Complement. Ther. Med..

[112] Devpura G (2021). Randomized placebo-controlled pilot clinical trial on the efficacy of ayurvedic treatment regime on COVID-19 positive patients. Phytomedicine.

[113] Afewerky HK (2020). Pathology and pathogenicity of severe acute respiratory syndrome coronavirus 2 (SARS-CoV-2). Exp. Biol. Med. (Maywood).

