# Supplementary material for: Sodium–calcium exchanger isoform-3 targeted Withania somnifera (L.) Dunal therapeutic intervention ameliorates cognition in the 5xFAD mouse model of Alzheimer’s disease
Source: Sci Rep. 2022 Jan 27;12:1537. doi: 10.1038/s41598-022-05568-2 (PMC8795410; doi:10.1038/s41598-022-05568-2)
Supplement: Supplementary file 1 — Supplementary Figures. [file 41598_2022_5568_MOESM1_ESM.doc]

**Sodium-calcium exchanger isoform-3 targeted *Withania somnifera* (L.) Dunal therapeutic intervention ameliorates cognition in the 5xFAD mouse model of Alzheimer’s disease**

Henok Kessete Afewerky^1, 2, 3, 4^, **^*^**, Hao Li^4, 5^, Tongmei Zhang^4, 5^, Xinyan Li^4, 5^, Yacoubou Abdoul Razak Mahaman^2, 4^, Limin Duan^4, 5^, Pengwei Qin^4, 5^, Jiequn Zheng^4, 5^, Lei Pei^1, 4^, Youming Lu^1, 4, 5^, **^*^**

^1^Department of Neurobiology, School of Basic Medicine, Tongji Medical College, Huazhong University of Science and Technology, Wuhan, China

^2^Department of Pathology and Pathophysiology, School of Basic Medicine, Tongji Medical College, Huazhong University of Science and Technology, Wuhan, China

^3^School of Allied Health Professions, Asmara College of Health Sciences, Asmara, Eritrea

^4^The Institute for Brain Research, Collaborative Innovation Center for Brain Science, Huazhong University of Science and Technology, Wuhan, China

^5^Department of Physiology, School of Basic Medicine, Tongji Medical College, Huazhong University of Science and Technology, Wuhan, China

**^*^**Corresponding authors: -

Henok Kessete Afewerky [henokessete@hust.edu.cn](mailto:henokessete@hust.edu.cn) and Youming Lu [lym@hust.edu.cn](mailto:lym@hust.edu.cn)

Department of Neurobiology, School of Basic Medicine, Tongji Medical College, Huazhong University of Science and Technology, Wuhan, China

**Supplementary Figures**

**Supplementary Figure S1:** Body weights (in grams) during the daily for 45-days of treatments at the mice age of 5-11 weeks. The data represent the mean ± SEM. The data were obtained from 12 independent mice in every cohort (n=12) applying ANOVA. No statistically significant difference was observed between the groups during every week of these periods of record (*p*>0.05). *Vehicle* control group, *LDT* low dose intervention, *HDT* high dose intervention, *Res* Resveratrol


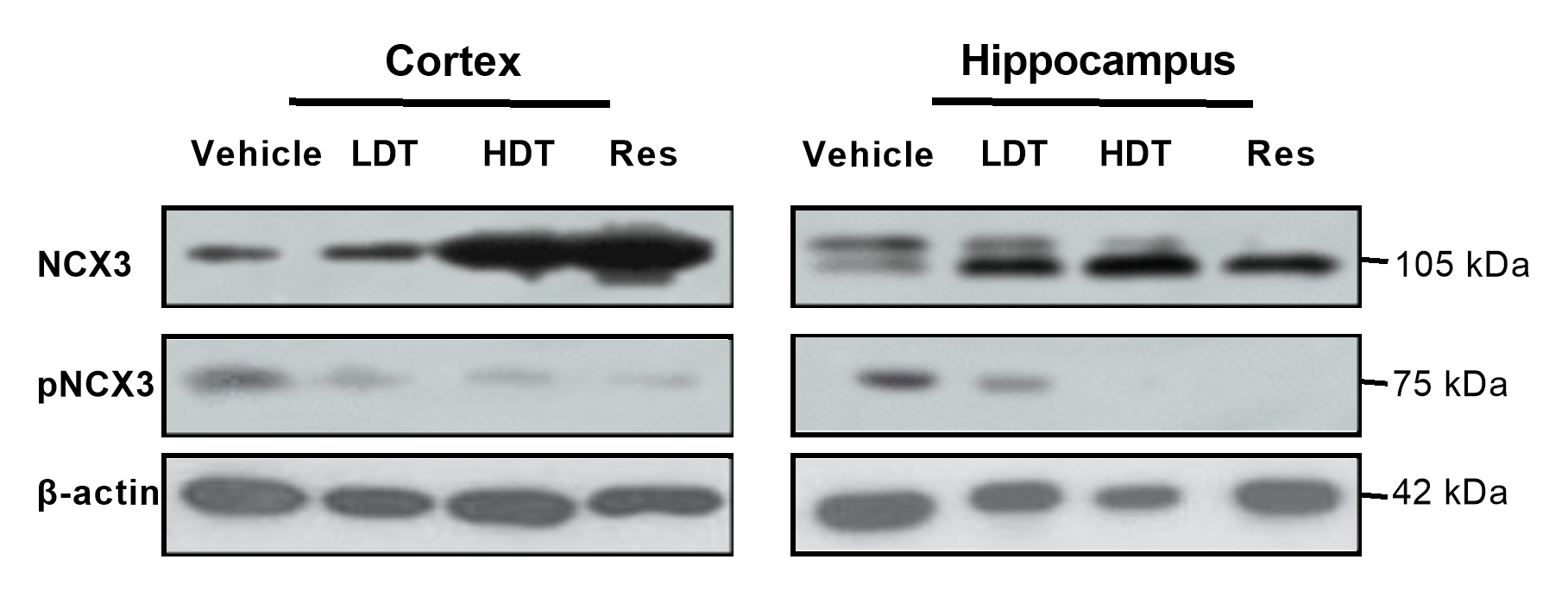


**Supplementary Figure S2:** NCX3 expression normalized based on a β-actin loading control from the same immunoblot. The composition of these panels is identical with Figure 3B. *NCX3* Na^+^-Ca^2+^ exchanger isoform 3, *pNCX3* proteolytic fragment of NCX3, *Vehicle* control group, *LDT* low dose treatment, *HDT* high dose treatment, *Res* Resveratrol


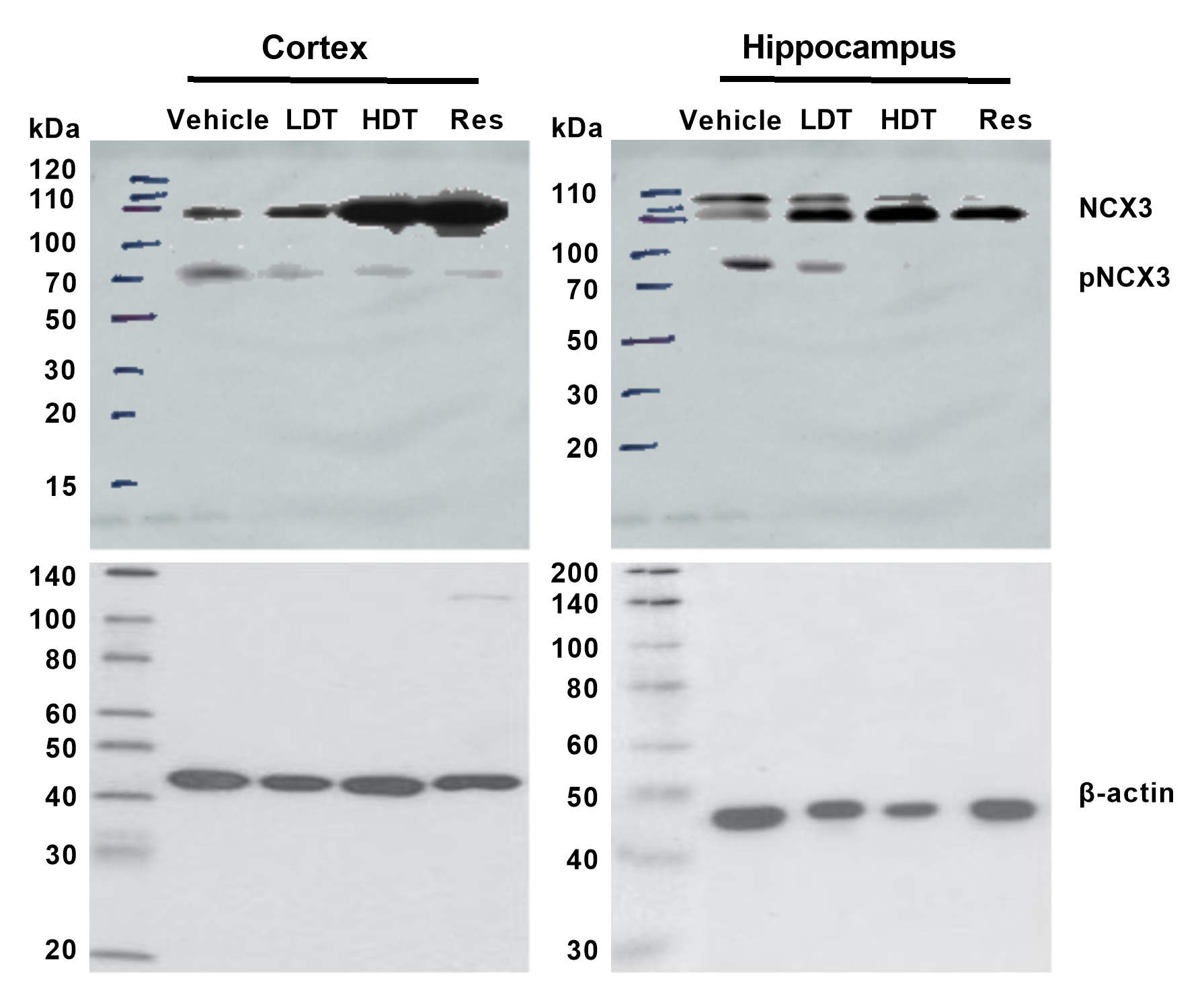


**Supplementary Figure S3:** Full-membrane images of NCX3 immunoblots presented in Figure 3B and Supplementary Figure S2. *NCX3* Na^+^-Ca^2+^ exchanger isoform 3, *pNCX3* proteolytic fragment of NCX3, *Vehicle* control group, *LDT* low dose treatment, *HDT* high dose treatment, *Res* Resveratrol


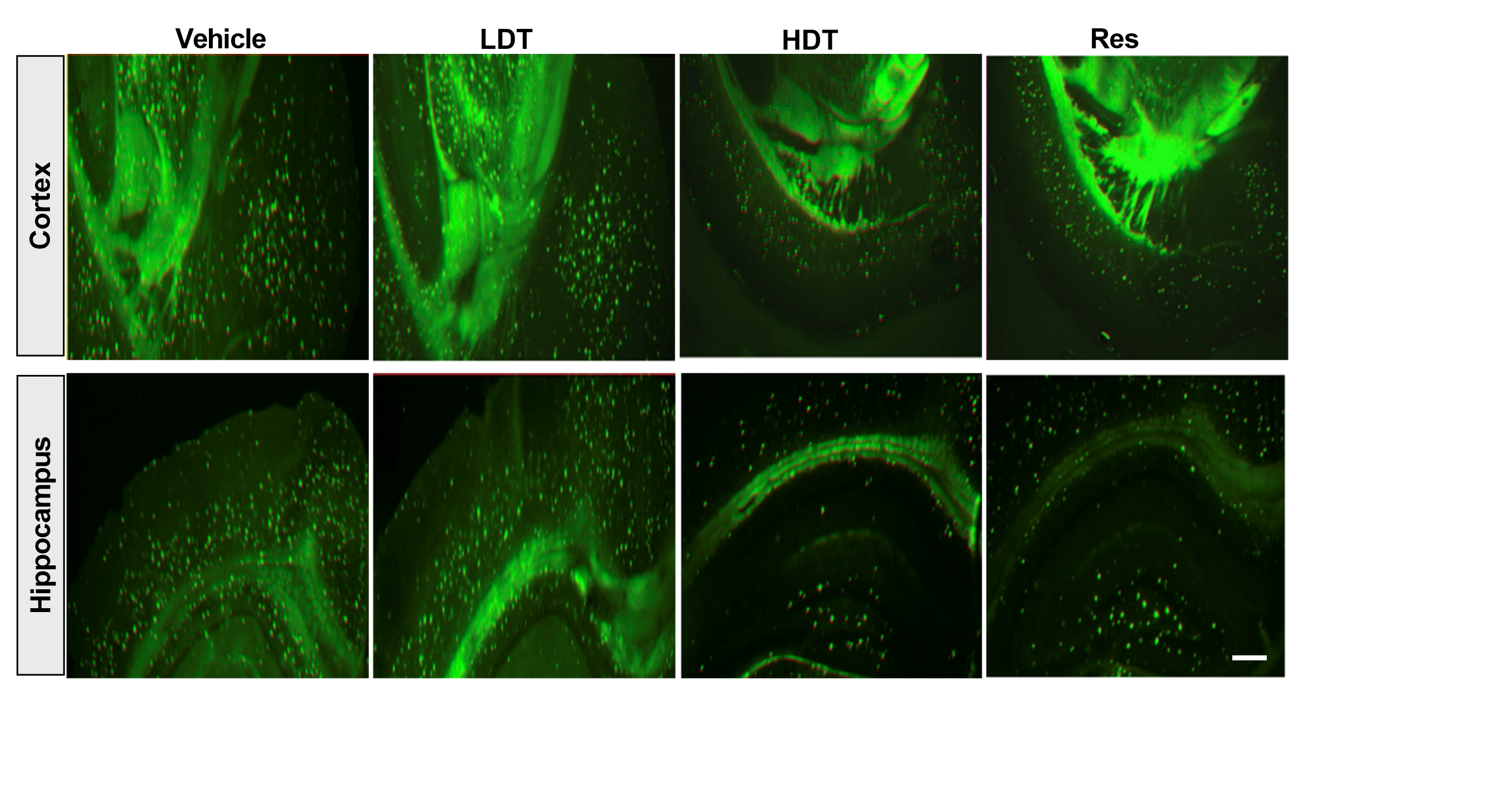


**Supplementary Figure S4:** A summary confocal microscope immunoreactive studies of Figure 4 and 5 (Scale bar = 500 μm). Aβ plaque deposition in the cortex and hippocampus region of 4-month-old 5xFAD mice brain tissue following 45 daily (P30-75) intervention using *W. somnifera* roots methanolic extract visualized with Thioflavin-S staining. *Vehicle* control group, *LDT* low dose intervention, *HDT* upper dose intervention, *Res* Resveratrol
